# Atypical core-periphery brain dynamics in autism

**DOI:** 10.1162/netn_a_00181

**Published:** 2021-04-27

**Authors:** Dipanjan Roy, Lucina Q. Uddin

**Affiliations:** Cognitive Brain Dynamics Lab, National Brain Research Centre, Manesar, India; Department of Psychology, University of Miami, Coral Gables, FL, USA

**Keywords:** Core-periphery dynamics, Atypical timescales, Caudate, Core and contextual symptom severity, Sensory-motor network, Restricted and repetitive behaviors

## Abstract

The intrinsic function of the human brain is dynamic, giving rise to numerous behavioral subtypes that fluctuate distinctively at multiple timescales. One of the key dynamical processes that takes place in the brain is the interaction between core-periphery brain regions, which undergoes constant fluctuations associated with developmental time frames. Core-periphery dynamical changes associated with macroscale brain network dynamics span multiple timescales and may lead to atypical behavior and clinical symptoms. For example, recent evidence suggests that brain regions with shorter intrinsic timescales are located at the periphery of brain networks (e.g., sensorimotor hand, face areas) and are implicated in perception and movement. On the contrary, brain regions with longer timescales are core hub regions. These hubs are important for regulating interactions between the brain and the body during self-related cognition and emotion. In this review, we summarize a large body of converging evidence derived from time-resolved fMRI studies in autism to characterize atypical core-periphery brain dynamics and how they relate to core and contextual sensory and cognitive profiles.

## INTRODUCTION

### Sensory Processing in Autism

Perhaps the most remarkable feature of autism spectrum disorder (ASD) is profound behavioral diversity across different individuals, which pertains to all factors involved in interactions with the physical and social environment (Baron-Cohen, Ashwin, Ashwin, Tavassoli, & Chakrabarti, 2009; Blakemore, Burnett, & Dahl, 2010; Bolton, Morgenroth, Preti, & Van De Ville, 2020; Lawson et al., 2015; Robertson & Baron-Cohen, 2017; Shafritz, Dichter, Baranek, & Belger, 2008; Uddin, 2021). This diversity underlies variability in personality, physiology, and mental capacity, which in turn are sculpted by not only complex biological influences (e.g., medication, genetic and epigenetic factors) but also various sociocultural factors (e.g., multilingual environments, social learning, trauma; Baron-Cohen et al., 2009; Baum et al., 2017; Bolton et al., 2020; Robertson & Baron-Cohen, 2017; Uddin, 2021). Developmental research suggests that sensory symptoms manifest early in life, and contribute unique variance to the diagnostic criteria of autism (Andreae, 2019; Chen, Nomi, Uddin, Duan, & Chen, 2017; Ciarrusta et al., 2019; Courchesne, Campbell, & Solso, 2011; Uddin, 2021). Neuroimaging evidence suggests that sensory symptoms originate from differences in low-level processing in sensory-dedicated regions in the autistic brain, and offers insight into circuit-level alterations (Abbott et al., 2018; Alaerts et al., 2015; Alaerts, Swinnen, & Wenderoth, 2016; Alaerts et al., 2014; Anderson et al., 2011; Baum et al., 2017; Collignon et al., 2013; Courchesne et al., 2011; Uddin, 2021). Studying the brain at rest has demonstrated that although the environment has an influence, the brain operates intrinsically and is modulated by, rather than controlled by, the external world (Baum et al., 2017; Bolton et al., 2020; Liégeois et al., 2019; Lin et al., 2016; Liu, Liao, Xia, & He, 2018). This modulation is a recursive process between the brain and the environment mediated by perception and action (Friston, 2009; Kiebel, Daunizeau, & Friston, 2008). This process is highly dynamic, as are both the environment and the brain (Bolton et al., 2020; Friston, 2009; Huang et al., 2015).

Individuals with severe autism usually have intellectual impairments and develop little spoken language. There are subgroups of autistic individuals who may have average or above-average IQ, but who still struggle with more subtle aspects of communication, such as body language (Jasmin et al., 2019; Ostrolenk, Bao, Mottron, Collignon, & Bertone, 2019; Robertson & Baron-Cohen, 2017; Supekar et al., 2013; Uddin, 2021; Uddin et al., 2015). In addition to social difficulties, many individuals with autism show restricted and repetitive behaviors (RRB) and sensory abnormalities (SA), and have narrow interests (Huang et al., 2015; Jao Keehn et al., 2019; Jao Keehn et al., 2017; Jasmin et al., 2019; Kana, Keller, Minshew, & Just, 2007; Manning, Tibber, Charman, Dakin, & Pellicano, 2015; Mash, Reiter, Linke, Townsend, & Müller, 2018; McKinnon et al., 2019; Mottron, Belleville, Rouleau, & Collignon, 2014; Moul, Cauchi, Hawes, Brennan, & Dadds, 2015; Robertson & Baron-Cohen, 2017; Uddin, 2021).

The brain as a whole shows less coordinated activity in autism, and one way of classifying subtypes that can include hyper- and hyporeactivity to sensory environments or unusual interest in sensory aspects of the environment could be to use brain network-based classification methods (Harlalka, Bapi, Vinod, & Roy, 2018, 2019; Nomi, Bolt, Ezie, Uddin, & Heller, 2017; Uddin et al., 2013). There is a growing body of evidence further suggesting that individual brain regions work in a less cohesive manner in autism, with widely distributed timescales and hierarchical organization of brain networks (Atasoy, Donnelly, & Pearson, 2016; Chaudhuri, Knoblauch, Gariel, Kennedy, & Wang, 2015; Gollo, Roberts, & Cocchi, 2017; Gollo, Zalesky, Hutchison, van den Heuvel, & Breakspear, 2015; Harlalka et al., 2019; Hasson, Yang, Vallines, Heeger, & Rubin, 2008; Hong et al., 2019; Kumar et al., 2016; Lin et al., 2016; London, 2018; Nomi, Bolt, et al., 2017; Nomi & Uddin, 2015; Nomi, Vij, et al., 2017; Oldham & Fornito, 2019; Pillai & Jirsa, 2017; Preti & Van De Ville, 2019; Raut, Snyder, & Raichle, 2020; P. Wang et al., 2019; S. Wang et al., 2015; Watanabe & Rees, 2017; Watanabe, Rees, & Masuda, 2019). Here we use the terms core and periphery (outside the core) brain regions when we refer to atypical timescales, flexibility, cohesion, dispersion, and functional gradients based on their hierarchical organization and differences in network dynamics (Chaudhuri et al., 2015; Gollo, 2019; Gollo et al., 2017; Gollo et al., 2015; Hasson et al., 2008; Hong et al., 2019; van den Heuvel, Kahn, Goñi, & Sporns, 2012; P. Wang et al., 2019; S. Wang et al., 2015). Specifically, studies have suggested a distinction between a network periphery containing sensory and motor regions with more locally clustered connectivity, and a rich-club “core” that aggregates long-range connections and serves as a backbone for transmodal integration, giving rise to behavior and cognition (Deco, Kringelbach, Jirsa, & Ritter, 2017; Gollo, 2019; Gollo et al., 2017; Gollo et al., 2015; Griffa & van den Heuvel, 2018; Harlalka et al., 2019; Hasson et al., 2008; Hilgetag & Goulas, 2020; Hong et al., 2019; Lin et al., 2016; Rashid et al., 2018; Shafritz et al., 2008; P. Wang et al., 2019; Watanabe & Rees, 2017).

Perturbation of resting-state brain dynamics and distortion of timescales of sensory-processing regions in individuals with autism compared with that of people without autism may shed light on core and contextual neural processing and links with symptom severity in the disorder (Andreae, 2019; Baron-Cohen et al., 2009; Cerliani et al., 2015; Foxe et al., 2015; Harlalka et al., 2019; Hasson et al., 2008; Henry, Dichter, & Gates, 2018; Jao Keehn et al., 2019; Jao Keehn et al., 2017; Jasmin et al., 2019; Mash et al., 2018; McKinnon et al., 2019; Mottron et al., 2014; Moul et al., 2015). Recent functional connectivity studies in autism further highlight two classes of relationships between functional connectivity among various brain regions during core (state invariant) versus contextual (state-dependent) neural processing (Jasmin et al., 2019; Robertson & Baron-Cohen, 2017).

**Figure 1.**
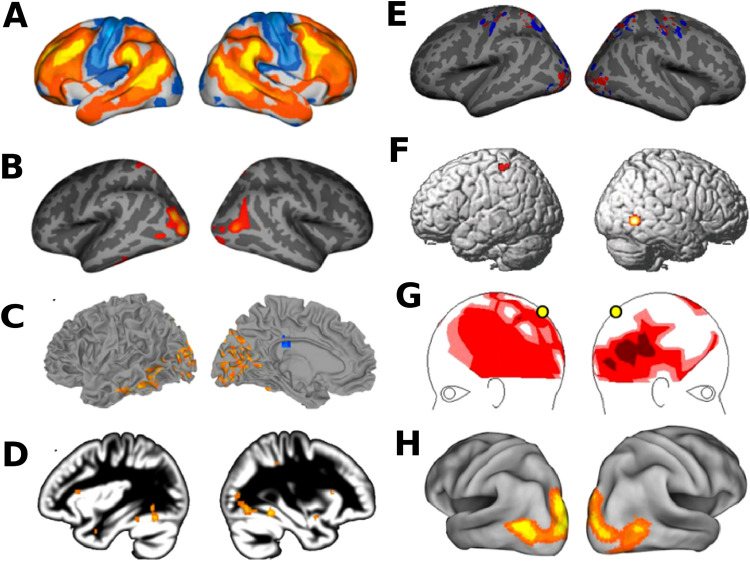
Interindividual variability and functional connectivity between autistic and neurotypicals in polymodal brain regions. (A) Interindividual variability in resting-state functional connectivity in neurotypical individuals. Positive and negative resting-state correlation values below the global mean value are displayed in warm and cool colors, respectively. (B) Regions showing greater cortical gyrification in autistic individuals compared with neurotypical individuals. (C) Regions of enhanced resting-state local connectivity are displayed, with greater connectivity in the autistic individuals than in neurotypicals (in warm colors), and regions of lower connectivity (in cool colors). (D) Regions showing greater activity in autistic individuals than in neurotypical individuals when processing visual stimuli (whole-brain FDR corrected). (E) The localization of the peak activation patterns is shown in autistic individuals (blue) and exhibits higher variability than in neurotypical individuals (red). (F) Clusters of brain structure alterations (differences in gray or white matter) between autistic and neurotypical individuals (whole-brain FDR corrected). (G) Regions where high MEG connectivity with the right parietal region (yellow circle, coherence analysis) is associated with high reading ability (darker color represents stronger correlation). (H) Regions of differences in multisensory activity between visually impaired and sighted individuals when processing auditory information (whole-brain FWE corrected). Figure adapted and modified with permission from Mottron et al. (2014).

Recent evidence further suggests that genetic mutations trigger brain reorganization in individuals with a low plasticity threshold in autism, mostly within sensory and extrasensory regions sensitive to cortical reallocations (Cheng, Rolls, Gu, Zhang, & Feng, 2015; Hahamy, Behrmann, & Malach, 2015; Mottron et al., 2014). These changes may account for the cognitive enhancements and reduced social expertise associated with autism (Mottron et al., 2014). Enhanced but normal plasticity may underlie non-syndromic autism, whereas syndromic autism may occur when a triggering mutation or event produces an altered plastic reaction, also resulting in intellectual disability and dysmorphism in addition to autism. Differences in the target of brain reorganization (perceptual vs. language regions) account for the main differences linking neocortical, cognitive, and genetic variability in autism that was proposed earlier as the trigger-threshold-target (TTT) model (Mottron et al., 2014). According to this model, the regions that are the most susceptible to reorganization in autism (the multimodal association regions) are also those that have the largest variability in terms of connectivity (structural and functional) among typical developing individuals (Figure 1A). The highest interindividual differences in resting-state connectivity are in the multimodal association cortex and the lowest are in the unimodal sensory and motor cortices (as displayed in Figures 1A and 1E). Furthermore, the same unimodal sensory regions, particularly lateral occipital cortex (LOC), exhibit greater gyrification index and volumetry in autistic individuals compared with typically developing individuals (as shown in Figures 1B and 1F). The same set of brain regions exhibits enhanced resting-state functional connectivity differences between autistic and typically developing individuals (Figure 1C) and high MEG connectivity based on coherence in the left parietal regions during visual processing (Figures 1G). Interestingly, the same set of brain regions also exhibits greater BOLD activity differences with neurotypicals during visual processing (as shown in Figures 1D), and the same set of brain regions are also involved in cross-modal plasticity in non-autistic, sensory-impaired individuals (Figures 1H; Mottron et al., 2014). This evidence of greater variability in the left visuo-motor superior parietal cortex and in the left associative visual areas in the autistic group compared with the control group, and idiosyncrasy over associative visual-motor areas and greater sensory functional connectivity, is prevalent throughout the autism literature (Alaerts et al., 2015; Alaerts et al., 2016; Alaerts et al., 2014; Foxe et al., 2015; Cerliani et al., 2015; Cheng et al., 2015; Ciarrusta et al., 2019; Hahamy et al., 2015; Harlalka et al., 2018; Henry et al., 2018; Keown et al., 2017; King et al., 2019; Lynch et al., 2013; Mash et al., 2018). In summary, the brain regions with largest cross-modal plasticity such as posterior superior temporal sulcus (pSTS), fusiform face area (FFA), precuneus, posterior cingulate cortex (PCC), and posterior parietal cortex (PPC), which are involved in perceptual processing, overlap with regions that are the most variable and most plastic in neurotypical individuals (Mottron et al., 2014). This overlap suggests a general mechanism for neuroplasticity, which mostly involves brain regions that are highly susceptible to reorganization. In contrast, primary sensory regions are less flexible and likely more hardwired, requiring a high degree of neural constraints because of their topographic (e.g., retinotopic/tonotopic) organization (Mottron et al., 2014).

Taken together, these findings suggest that the highest interindividual differences in resting-state connectivity are in the multimodal association cortex and the lowest are in the unimodal sensory and motor cortices, following a cortical hierarchy. This gradient of connectivity strength from early sensory areas to polymodal cortices may directly impact whole-brain dynamism and higher order multisensory integration in autism (Fu et al., 2019; Guo et al., 2017; Guo et al., 2020; Harlalka et al., 2019; Hong et al., 2019; King et al., 2019; Nair, Treiber,Shukla, Shih, & Müller, 2013; Noel, De Niear, Stevenson, Alais, & Wallace, 2017; Nomi &Uddin, 2015; Ostrolenk et al., 2019; Watanabe & Rees, 2017; Watanabe et al., 2019).

### Maturation of Core-Periphery Brain Networks in ASD: Structural Changes

Brain network maturation occurs as early as the neonatal stage, showing sharp increases between 6 and 9 years of age, then follows a protracted development throughout young adulthood, becoming largely mature by age 20; however, there are also many changes that continue to occur throughout adulthood and into old age (Ciarrusta et al., 2019; Courchesne et al., 2011; Foxe et al., 2015; Henry et al., 2018; Oldham & Fornito, 2019; Olson et al., 2020). Early brain volume overgrowth during infancy and the toddler years has been observed in autistic children, followed by an accelerated rate of decline in size and perhaps degeneration from adolescence to late middle age (Ciarrusta et al., 2019; Courchesne et al., 2011). Previous longitudinal and cross-sectional MRI studies reported maturation-related anatomical abnormalities in ASD, including overgrowth in early life but accelerated decline during adolescence and young adulthood (Abbott et al., 2018; Alaerts et al., 2014; Anderson et al., 2011). The frontal lobe, implicated in functions with high social and executive demands, showed the most severe enlargement in ASD beginning between 2 and 3 years of age, and frontal gray matter developed at an atypical growth rate in children with ASD (Anderson et al., 2011). Cortical thickness studies also clarified abnormal longitudinal neurodevelopmental trends with regional specificity in individuals with ASD, suggesting that cortical development in ASD first undergoes an expansion at a high rate in early childhood, then undergoes accelerated thinning until adolescence, and finally protracted thinning in early adulthood (Abbott et al., 2018; Anderson et al., 2011; Courchesne et al., 2011). Further, diffusion-weighted imaging (DWI) studies examined fractional anisotropy (FA), mean diffusivity, and structural connectivity (SC) using tractography to characterize various stages of the adult life span (Baum et al., 2017; Dajani et al., 2020; Huang et al., 2015). Volumetric studies suggest an early period of brain overgrowth in ASD followed by slowed growth during later childhood when the typically developing braincatches up with that of the autistic brain in terms of volume (Courchesne et al., 2011). Moreover, a recent study found that modular organization and small-world attributes are evident at birth, with several important topological metrics increasing monotonically during development (Huang et al., 2015). Most significant increases of regional nodes occur in the posterior cingulate cortex, a hub region of the default mode network (DMN). Positive correlations exist between nodal efficiencies and FA values of the white matter tracts, while correlations between efficiencies and FA values vary among many brain regions. These results reveal substantial topological reorganization of human brain structural networks through infancy and childhood, which is likely to be the outcome of both heterogeneous strengthening of the major white matter tracts and pruning of other axonal fibers. The anatomical connectivity alterations during early development and increased modular segregations between anatomical brain areas and large-scale brain networks mediate development of executive functions at youth (Baum et al., 2017). Interestingly, both cross-sectional and longitudinal data support the increased segregation-with-maturation and decrease thereafter story (Baum et al., 2017; Dajani et al., 2020; Huang et al., 2015; Keown et al., 2017; Lawson et al., 2015). These findings further set the stage for conducting more targeted investigation into multidimensional brain and behavioral links spanning neurodevelopmental processes that support executive functions, memory, and salience processing (Keown et al., 2017; Khambhati, Medaglia, Karuza, Thompson-Schill, & Bassett, 2018; Lawson et al., 2015; Liégeois et al., 2019; Preti & Van De Ville, 2019; Rosenthal et al., 2013; Xia et al., 2018).

### Maturation of Core-Periphery Brain Networks and Functional Connectivity

Emerging evidence suggests that ASD is associated with atypical trajectories of brain maturation (Guo et al., 2017). This has been indexed by decreased spontaneous low-frequency fluctuation (ALFF) of BOLD amplitude in the right precuneus and left middle occipital gyrus during all developmental stages (Guo et al., 2017). Significant diagnosis by age interactions is mediated by medial prefrontal cortex (mPFC), a key node of the DMN, with lowered ALFF in autistic children but higher ALFF in autistic adolescents and adults (Guo et al., 2017). More specifically, the quadratic changes of ALFF associated with increasing age in mPFC in neurotypicals were largely absent in ASD. Additionally, abnormal ALFF values in ASD-related brain regions predicted social deficits in ASD (Guo et al., 2017).

Recent functional connectivity evidence further suggests that in neonates with and without a family history of ASD, those with a family history had significantly higher neural activity in the right fusiform and left parietal cortex (Ciarrusta et al., 2019). In addition, the pattern of age-related changes in spontaneous activity in the cingulate and insula was disrupted in infants with a family history of ASD (Ciarrusta et al., 2019). Furthermore, stronger functional connectivity and the degree of overconnectivity between visual and sensorimotor networks were associated with greater autism symptoms in toddlers (B. Chen et al., 2020; Ciarrusta et al., 2019).

Significant hypoconnectivity has been observed in adolescents, especially in the DMN, while younger children exhibit both hyper- and hypoconnectivity (Harlalka et al., 2018). Furthermore, few recent studies highlight the importance of age stratification to test the developmental hypothesis that hyperconnectivity of brain networks may be more characteristic of young children with ASD, while hypoconnectivity may be more prevalent in adolescents and adults (Harlalka et al., 2018, 2019; Henry et al., 2018; Nomi & Uddin, 2015). In one study, the authors found that in the youngest cohort (age 11 and under), children with ASD exhibited hyperconnectivity within large-scale brain networks including the DMN, salience network (SN), and executive control network (ECN) as well as decreased between-network connectivity compared with age-matched neurotypicals. In contrast, adolescents with ASD (in the age range 11–18) did not differ from neurotypicals in within-network connectivity, yet showed decreased between-network connectivity compared with neurotypicals (Nomi & Uddin, 2015). Adults with ASD showed no within- or between-network significant differences in functional network connectivity compared with age-matched controls (Nomi & Uddin, 2015). Taken together, atypical development of functional connectivity patterns in key sensory and higher order multimodal brain regions may index vulnerability for autism.

Many previous cross-sectional functional connectivity studies reported that individuals with ASD exhibited atypical developmental trajectories of DMN connectivity and frontostriatal connectivity across childhood and adolescence, and a significant interaction between diagnosis and age was observed in several core DMN regions, such as the mPFC, PCC, and precuneus (Cheng et al., 2015; Delmonte, Gallagher, O’Hanlon, McGrath, & Balsters, 2013; Guo et al., 2017; Harlalka et al., 2018; Henry et al., 2018). Functional connectivity circuits of the pSTS, a core hub region implicated in sociocognitive processing, has also been shown to exhibit atypical developmental trajectories in ASD (Alaerts et al., 2015; Alaerts et al., 2016).

Research examining developmental changes in large-scale network functional connectivity demonstrated that individuals with ASD exhibited different abnormal patterns of within- and between-network connectivity during different developmental stages (Guo et al., 2017; Harlalka et al., 2018; Henry et al., 2018; Nomi & Uddin, 2015). In spite of site-specific heterogeneity and inclusion of nonoverlapping samples in the studies listed in Table 2 from the Autism Brain Imaging Data Exchange (ABIDE), there is increasing convergence in support of a developmental model accounting for the age-specific over- and underconnectivity findings in ASD. This model posits that childhood autism is characterized by brain hyperconnectivity, whereas adolescent and adulthood autism is characterized by brain hypoconnectivity (B. Chen et al., 2020; Delmonte et al., 2013; Guo et al., 2017; Guo et al., 2019; Harlalka et al., 2018; Henry et al., 2018; Huang et al., 2015; Nomi & Uddin, 2015). These findings suggest atypical cortical developmental trajectories across the life span, and highlight the importance of taking different developmental stages into account when exploring the potential neural mechanisms of ASD (Gollo et al., 2017; Hahamy et al., 2015; Henry et al., 2018; Naik, Banerjee, Bapi, Deco, & Roy, 2017; Naik, Subbareddy, Banerjee, Roy, & Bapi, 2017).

Another interesting recent study evaluating age and gender effects jointly on intrinsic functional connectivity found that the ASD group was characterized by an increase in regional segregation into distinct functional networks, followed by a marked decrease in segregation across time. The opposite pattern was observed for neurotypical individuals, suggesting that segregation of functional networks persists into adulthood in typical development but not in ASD (Henry et al., 2018). A similar pattern was observed in integration results: Individuals with ASD evidenced increased functional integration over development, whereas the opposite was observed for typically developing (TD) individuals. These findings help to clarify prior work that showed hypoconnectivity in ASD for adults within various large-scale brain networks (DMN, SN, ECN), as well as between networks (Kana et al., 2007; Lynch et al., 2013; Supekar et al., 2013; Uddin et al., 2013), whereas the opposite has been found for children with ASD (Anderson et al., 2011; Guo et al., 2020; Harlalka et al., 2018, 2019). However, complementary to the above findings, it was also observed that there was an overall idiosyncratic pattern distributed over the whole brain in adults with ASD, with a lack of segregation of functional networks and a higher integration of the component regions of interest in ASD (Hahamy et al., 2015). Thus, hypoconnectivity within networks may partially be explained by a lack of differentiation in adulthood and increased connectivity within sensory areas (B. Chen et al., 2020).

**Figure 2.**
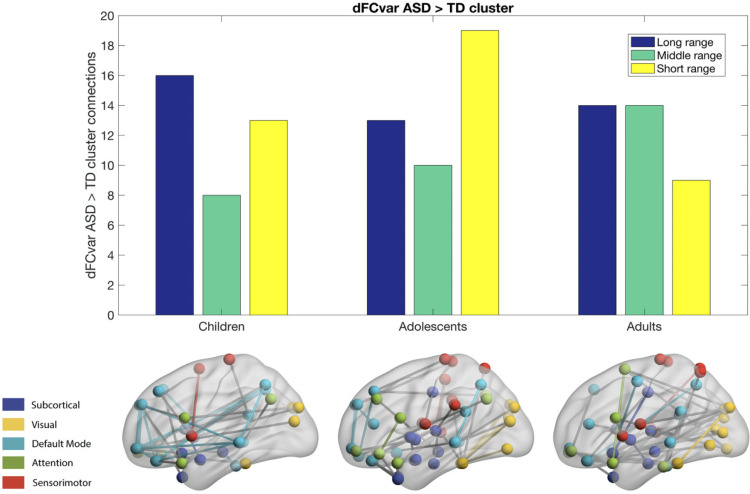
Hypervariant ASD connections estimated using dFCVar matrix. The majority of connections in children are long-range, while the adults exhibit hypervariability in dFC in both middle-range and long-range connections. Adolescents are seen to have majority short-range connections exhibiting hypervariability. Figure adapted with permission from Harlalka et al. (2019).

**Figure 3.**
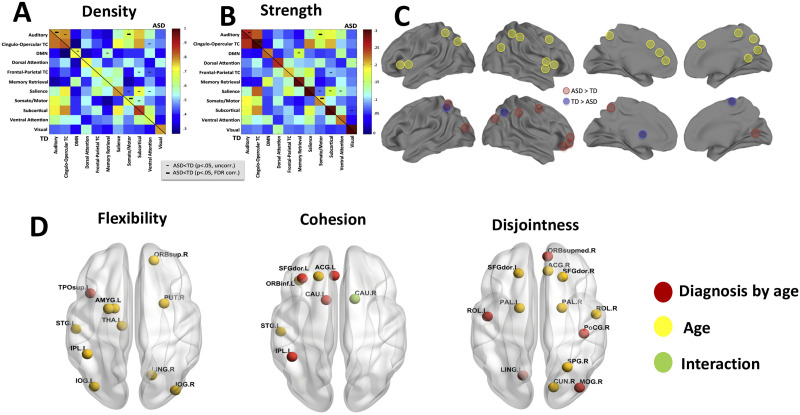
Globally atypical network flexibility of brain modules in autism. (A, B) Connection density (A) and strength (B) between each pair of networks. Group averages are shown for the TD group in the lower left triangle and for the ASD group in the upper right triangle. Network connections with lower density or strength are denoted by dashes (bold indicates *p* < 0.05 after FDR correction). (C) Approximate location of nodes with highest betweenness centrality in the TD group (yellow), and nodes with greater (red) or reduced (blue) betweenness centrality in ASD (all *p* < 0.05, uncorrected). (D) Brain plot of areas showing significant effect of age, diagnosis by age, and interaction effect on flexibility, cohesion strength, and disjointness, respectively. Typically developing, TD; autism spectrum disorder, ASD. Figure adapted with permission from Harlalka et al. (2019) and Keown et al. (2017).

There are very few studies that have examined the relationship between age-related change in intrinsic functional connectivity and gender in ASD and TD (Guo et al., 2017; Harlalka et al., 2018; Henry et al., 2018). The majority of resting-state fMRI studies of autism have focused on characterizing intrinsic large-scale brain network organization in adolescent and adult males, barring a few studies that have given some consideration to both age and gender (Guo et al., 2017; Harlalka et al., 2018; Henry et al., 2018; Lai et al., 2017). A large majority of these studies found that ASD exhibits increased functional integration at the expense of decreased functional segregation (Abbott et al., 2018; Alaerts et al., 2015; Alaerts et al., 2016; Alaerts et al., 2014; Cerliani et al., 2015; Cheng et al., 2015; Delmonte et al., 2013; Harlalka et al., 2018; Henry et al., 2018; Keown et al., 2017). In adolescents with ASD, there is a significant decrease in modularity, suggesting a less robust modular organization, and an increase in participation coefficient, suggesting more random integration and widely distributed connection edges (Harlalka et al., 2018; Henry et al., 2018; Keown et al., 2017). Modularity decreased nonlinearly in the ASD group with age, as evidenced by an increase and then a decrease over development. Age effects on modularity were localized to the somatosensory network (Henry et al., 2018). Furthermore, there is significant hypoconnectivity observed in the adolescent group, especially in the DMN (Harlalka et al., 2018), while children showed both hyper- and hypoconnectivity (Harlalka et al., 2018; Henry et al., 2018). While the findings were applied at the global level, they were not equally robust across all networks and in one case (i.e., greater cohesion within the ventral attention network in ASD) even reversed (Keown et al., 2017; shown in Figures 2 and 3). Among those studies that investigated gender effects in an age-constrained manner was one reporting that TD female individuals had higher quadratic effects of age on modularity that trended toward significantly different from male individuals with ASD, and males and females showed a differential neural expression of ASD, characterized by predominantly hypoconnectivity patterns in males with ASD (compared with TD males), and hyperconnectivity in females with ASD (compared with TD females; Alaerts et al., 2016; Harlalka et al., 2018; Henry et al., 2018). As higher order interaction and nonlinear regression effects are notoriously underpowered even for large sample sizes, these results in terms of reduced age and gender-specific trends in differentiation and functional specialization of brain networks should be interpreted with caution (Courchesne et al., 2011; King et al., 2019).

Efficient functioning of specialized sensorimotor and cognitive networks relies on two complementary organizing principles: functional segregation (or differentiation), emphasizing the degree to which different regions or networks are specialized, and functional integration, referring to the communication between regions within a specialized network (Abbott et al., 2018; Anderson et al., 2011; Harlalka et al., 2018; Henry et al., 2018; Keown et al., 2017; Nomi & Uddin, 2015; Padmanabhan, Lynch, Schaer, & Menon, 2017; Ray, Hajare, Roy, & Banerjee, 2020; Reiter et al., 2019). The differential relationship between modularity and age seen in ASD was in a large part due to the peripheral networks (somato-sensorimotor and visual networks). This result from localization analysis suggests that the somatosensory network drives, at least in part, the increase in modularity across time seen in neurotypicals relative to those with ASD (Harlalka et al., 2018; Henry et al., 2018). In a recent study, it was further demonstrated in toddlers with ASD that the degree of overconnectivity between visual and sensorimotor networks was associated with greater autism symptoms, and an age-related weakening of the visual-auditory between-network connectivity was observed in the ASD but not the TD group (B. Chen et al., 2020).

Thus, differentiation and specification of regions related to the visual and somato-sensorimotor network appears to contribute greatly to functional connectivity changes across development. However, a network knockout approach was used to isolate the influence of specific functional networks, a simple leave-one-out process demonstrating that the somatosensory cortex had no effect on global efficiency models (Harlalka et al., 2018; Henry et al., 2018; Keown et al., 2017), which suggests that the differences in global efficiency between ASD and TD were not localized to the somatosensory cortex, but rather reflect a more global whole-brain phenomenon (Henry et al., 2018; Keown et al., 2017). Longitudinal studies demonstrate that childhood executive functions largely predict variance in autistic individuals’ adaptive behavior later in life (Kenny et al., 2019). Taken together, this work highlights the need for more targeted future research and investigation of the brain mechanisms at various stages of maturation in both male and female ASD to pinpoint subtypes of functional connectivity patterns across development linking adaptive behavior, cognitive flexibility, executive task processing, and manifestations of core and contextual deficits in ASD across the life span.

### Sensory and Sociocognitive Deficits in ASD: Behavior and Neuroimaging Studies

Studies of neurotypical individuals and those with ASD have typically utilized a variety of behavioral paradigms ranging from sensory-motor perceptual integration, attention, cognitive flexibility and executive functions, face-to-face communication, and conversation, all of which can be broadly categorized as (a) tasks with high social and executive demands (Jasmin et al., 2019), and (b) tasks with high sensory but low social demand (Robertson, Martin, Baker, & Baron-Cohen, 2012).

Here, we review a sampling of behavioral studies in which individuals with autism display sensory and cognitive deficits (see Table 1 for a non-exhaustive list of examples). More specifically, we have reviewed tasks with greater sensory demands, overt sensory-motor, repetitive behavior (low social demands), examining core neural features originating from abnormal thalamic and striatal interactions and sensory input gating. We also highlight a sampling of studies with social components such as face-to-face communication, day-to-day conversation, emotions, pragmatics, and sarcastic prosody that are highly context dependent (high social demands) and used to examine contextual (state-dependent) neural features. Subcortical areas also play a crucial role in core neural processing as highlighted above; specifically, determining adaptive behavior, state- and trait-specific variability, and flexibility. However, only a handful of studies have looked at their potential role in symptom severity in autism. Several studies showed decreased functional connectivity among cortical regions associated with social functions, such as the superior temporal sulcus, medial prefrontal, temporoparietal junction, left inferior frontal gyrus, as well as somatosensory cortex (Alaerts et al., 2015; Alaerts et al., 2014; Anderson et al., 2011; Baum et al., 2017), with some showing simultaneously increased functional connectivity between thalamus, striatum, and some of the same cortical regions (Abbott et al., 2018; Alaerts et al., 2015; Anderson et al., 2011; Cerliani et al., 2015; but see Nair et al., 2013). Findings from the task-based functional magnetic resonance imaging literature demonstrate that the above subcortical and cortical brain regions are involved in restricted and repetitive behaviors (RRB) and may represent some of the earliest biomarkers of ASD (B. Chen et al., 2020; Ciarrusta et al., 2019; Robertson & Baron-Cohen, 2017). Previous studies also reported that individuals with autism showed increased functional connectivity between regions in the frontal cortex (anterior cingulate cortex, ACC; middle frontal gyrus, MFG; paracingulate gyrus, Pcg; and orbitofrontal cortex, OFC), and striatum (nucleus accumbens, NAcc; and caudate) compared with neurotypical individuals (Abbott et al., 2018; Cerliani et al., 2015; Delmonte et al., 2013; Jasmin et al., 2019).

**Table 1.** Sampling of studies examining restrictive and repetitive behavior, sensory abnormality, executive functions, communication, sociocognitive processing, and mentalizing in autism

**Behavioral paradigms**	**Type of stimuli**	**References**
**Sensory-motor perception and integration**		
Visual acuity (VA) and perception	Freiburg Visual Acuity and Contrast Test	Ashwin, Ashwin, Rhydderch, Howells, and Baron-Cohen (2009)
Integration of sensory input and visual reaction time (RT)	Visual search task (Feature and conjunction type)	Plaisted et al. (1998)
Atypical visual saliency	Gaze patterns during natural scene-viewing	Pelphrey, Morris, and McCarthy (2005)
Integration of motion information	Directional variability in standard motion dot coherence task	Manning et al. (2015)
Integration of motion signals and perceptual decision	Motion discrimination task, manually indicating the global direction of motion in a random dot across a range of coherence level	Robertson, Martin, Baker, and Baron-Cohen (2012)
**Attention, cognitive flexibility, and executive functions**		
Motor inhibition, decision-making, and set switching	Three different EF tasks: (a) motor-inhibition (GO/NO-GO); (b) cognitive interference-inhibition (spatial STROOP); and (c) set shifting (SWITCH)	Schmitz et al. (2006)
Executive function (EF), response inhibition	Response inhibition task during alphabetic letter matching criterion under three experimental conditions	Kana et al. (2007)
Executive function (planning, inhibition, and cognitive flexibility) and theory of mind (false-belief understanding)	Longitudinal study 12-year follow-up Time 1 tested on components of executive function (planning, inhibition, and cognitive flexibility) and theory of mind (false-belief understanding). At Time 2, tested participants’ autistic features and adaptive behavior.	Kenny et al. (2019)
Behavioral inflexibility, attention and executive functions	Stimulus-evoked brain states involving performance of social attention and numerical problem-solving tasks	Uddin et al. (2015)
Cognitive shift, repetitive and restrictive behavior	A target detection task during which geometric shapes (squares, triangles, or circles) were presented one at a time. Participants were required to classify each stimulus as a “target” or “non-target” on the basis of its shape and respond with an appropriate button press.	Shafritz et al. (2008)
Metacognitive executive abilities and atypical flexibility	Using BRIEF: Behavior Rating Inventory of Executive Function scale to access behavior	Moul et al. (2015)
**Face-to-face communication, multisensory speech perception, sociocognitive processing, and mentalization**		
Sociocognitive response and communication	Face-to-face structured and unstructured communication using a modified version of the Interest Scale questionnaire	Jasmin et al. (2019)
Multisensory processing (audio-visual Integration)	Synchronous auditory pip during a complex visual search task (pip-pop effect)	Collignon et al. (2013)
Multisensory facilitation using sensory integration	Nonsocial stimuli (i.e., flashes and beeps)	Ostrolenk et al. (2019)
Eye gaze to integrate joint role of attention and comprehension of mental states of others	Stimulus presentation is based on congruent and incongruent trials over which participant needs to integrate information to comprehend what a virtual actor ought to do in a given context. Social and contextual stimuli.	Plaisted et al. (1998)
Atypical cross-modal (auditory-visual) modulation linked to sociocommunicative deficits	Auditory (high or low pitch) and visual conditions (dot located high or low in the display) were presented, and participants indicated whether the stimuli were “high” or “low”	Jao Keehn et al. (2017)
Atypical audio-visual temporal recalibration and speech stimuli	Asynchronous audio-visual stimuli of varying levels of complexity and performance of a simultaneity judgment (SJ)	Noel et al. (2017)
Intelligible multisensory speech perception	Integrated seen and heard speech were accessed while the environmental noise was systematically manipulated	Foxe et al. (2015)

Furthermore, increased functional connectivity between ACC and caudate was associated with reduced activation to social rewards in the caudate (Delmonte et al., 2013). Greater connectivity between the right MFG and caudate was associated with higher RRBs, and connectivity between the bilateral Pcg and NAcc, and the right OFC and NAcc, was negatively associated with social and communicative deficits (Cerliani et al., 2015; Delmonte et al., 2013). These findings indicate that abnormalities in frontostriatal circuitry potentially underlie major deficits in ASD, social interaction and communication difficulties and RRBs (Alaerts et al., 2015; Delmonte et al., 2013; Guo et al., 2020; Guo et al., 2019; Jasmin et al., 2019). Neuroimaging evidence further demonstrates that autistic adolescents show atypical activation of the mentalizing system. Longitudinal follow-up of a group of autistic children who passed or failed theory of mind tests revealed reduced activation of the medial prefrontal cortex and precuneus, posterior cingulate and lateral temporal cortices in children, and predicted significant variance in young children’s adaptive behavior at the 12-year follow-up (Kenny et al., 2019).

Although the development of co-occurrence of deficits in sensory and higher order sociocognitive processing in ASD is a topic of considerable interest, links between core-periphery brain network maturation and how they contribute to behavioral variability and unique variance to the diagnostic criteria of autism are not yet firmly established. However, the studies reviewed here provide critical insights into atypical integration of sensory input at the local level, eventually leading to impairment manifested at the global level, addressing simultaneity of sensory and as well as sociocognitive deficits encountered in autistic children, adolescents, and adults.

### Neural Substrates of Behavioral Variability in Autism: Predictions From Atypical Core-Periphery Dynamics

One paradox of autism is the co-occurrence of deficits in sensory and higher order sociocognitive processing (Hong et al., 2019). Here, we review whether these phenotypic patterns based on different brain dynamics approaches (flexibility of system-level transitions, functional gradients, and timescale hierarchy) all converge in relating overarching system-level imbalance—specifically a disruption in macroscale hierarchy affecting integration and segregation of core polymodal and peripheral unimodal networks. Task-based neuroimaging is necessary to understand the neural basis of atypical sensory and cognitive processing in several domains such as face-to-face conversation, working memory, attention, executive processing, and perception; however, given the decreased cognitive demands and potential for reuse, resting-state functional magnetic resonance paradigms or task-free approaches in autism have become a promising avenue for discovery in recent years. Resting-state paradigms are also adoptable with some degree of ease by various multimodal approaches (EEG, MEG, ECOG) for generating specific hypotheses based on characterizing normative brain dynamical patterns (Chang & Glover, 2010; Sahoo, Pathak, Deco, Banerjee, & Roy, 2020). Moving forward, one could use resting-state fMRI for precise quantification of typical and atypical flexibility based on core-periphery interactions (highly connected hub regions and small brain regions located outside the core regions; e.g., striatum, subcortical areas) to discover whether atypical processing in peripheral brain regions potentially gives rise to perturbation of large-scale macroscale brain network dynamics (Gollo et al., 2017; Guo et al., 2020; Harlalka et al., 2019; Hong et al., 2019; Preti & Van De Ville, 2019; Rashid et al., 2018; Vidaurre, Smith, & Woolrich, 2017; Watanabe & Rees, 2017; Watanabe et al., 2019). Beyond revealing brain regions activated in response to specific task conditions, resting-state functional connectivity approaches permit analysis of how cognitive functions emerge from precise timing and concerted activity in the specialized large-scale brain network interactions (Harlalka et al., 2019; Nomi, Vij, et al., 2017; Preti & Van De Ville, 2019; Vidaurre et al., 2017; Watanabe & Rees, 2017; Watanabe et al., 2019). Dynamic functional connectivity (dFC) approaches further enable the study of moment-to-moment variability in neurotypical and autistic individuals, as documented by several recent studies (see Table 2). Furthermore, dFC variability is quantified by the standard deviation of time-varying dynamic functional connectivity. Hence, dFCVar tracks the changes in variability in dynamic functional connectivity between brain regions anchored in large-scale neurocognitive networks. These measures are now frequently used to characterize atypical hyper- and hypofunctional connectivity variability in neurodevelopmental disorders (H. Chen et al., 2017; Liégeois et al., 2019; Gollo et al., 2017; Guo et al., 2020; Nomi, Bolt, et al., 2017; Nomi, Vij, et al., 2017). A comprehensive review of various dynamic functional connectivity methods and their application in psychopathology and flexible behavior is available elsewhere (Bolton et al., 2020; Uddin, 2021).

**Table 2.** Sampling of fMRI studies capturing atypical core-periphery brain dynamics and relationships with symptom severity

**ASD and TD samples**	**Age range (mean, *SD*)**	**Analysis pipeline**	**Reference**
ASD: 79 (included 31 out of 79) TD: 105 (included 44 out of 105) (ABIDE I)	ASD: 7–18 (not including 18 years) (mean: 12.46, *SD*: 3.1) TD: 7–18 (mean: 11.51, *SD*: 2.64)	dFCVar estimate using time-varying functional connectivity of seven subnetworks composed of subcortical (SC), auditory (AU), visual (VIS), somatomotor (SM), cognitive control (CC), default mode (DM), and cerebellar (CB) networks. To determine the connectivity states, covariance matrices of ASD and TD were clustered by k-means clustering algorithm based on Manhattan distance. Clustered centroid matrices were covariance matrices of connectivity states and their relationship with symptom severity.	Yao et al. (2016)
ASD: 24 TD: 26 (ABIDE Utah site primary, Indiana and Zurich site replication)	ASD: 18.4–38.9 (mean: 25.3, *SD*: 5.5) TD: 18.2–39.3 (mean: 25.3, *SD*: 6.3)	Energy-landscape analysis across seven well-established resting-state brain networks to characterize atypical neural state transition probability between core DMN, CEN, VAN, DAN, and peripheral sensory networks and to quantify relationship with symptom severity.	Watanabe and Rees (2017)
Total 507 male subjects ASD: 209 TD: 298 (all ABIDE sites)	ASD: 6–36 (mean: 16.5, *SD*: 6.2) TD: 6–36 (mean: 16.8, *SD*: 6.2)	Standard deviation (*SD*) of dFC (dFCVar) matrix to compute hypervariant connections across whole-brain regions of interest and relationship with symptom severity.	H. Chen et al. (2017)
TD and ASD children combined: 774; 560 with SRS ASD: 22 (ABIDE sites)	Combined: 6–10 (mean: 7.99, *SD*: 1)	Sliding-window correlation to estimate dFC and estimation of dwell time based on fractional occupancy (FO) index. Globally disconnected vs. hyperconnected whole-brain networks and core DMN hub network and relationship with symptom severity.	Rashid et al. (2018)
TD children: 28 ASD children: 29	ASD: 3–7 (mean: 4.99, *SD*: 1.32) TD: 3–6 (mean: 4.99, *SD*: 1.01)	*K*-means cluster analysis was performed to identify distinct temporal states based on the spatial similarity of each functional connectivity pattern. Estimation of dynamic functional connectivity variance (dFCVar) between the hub regions of the core DMN and sensory-motor network and relationship with symptom severity.	He et al. (2018)
ASD children: 26 TD children: 26 Adolescent ASD: 28 Adolescent TD: 28 Adult ASD: 18 Adult TD: 18 (ABIDE NYU site)	Child ASD: 7.15–10.06 (mean: 9.51, *SD*: 1.12) Child TD: 6.47–10.86 (mean: 9.10, *SD*: 1.32) Adolescent ASD: 11.01–17.88 (mean: 13.71, *SD*: 1.79) Adolescent TD: 11.32–16.93 (mean: 14.01, *SD*: 1.74) Adult ASD: 18.58–39.1 (mean: 24.13, *SD*: 3.92) Adult TD: 18.59–31.78 (mean: 25.41, *SD*: 5.87)	Sliding-window analysis to calculate variability of dFC (dFCVar) in order to quantify proportion of short-range, long-range hypo- and hyperconnectivity (in each age group) patterns in core-periphery brain networks composed of visual, sensorimotor, subcortical, DMN, attention (identified using multilayer modularity detection algorithm). Quantification of atypical flexibility, cohesiveness, and disjointness of core hub regions and peripheral brain regions and relationship with symptom severity.	Harlalka et al. (2019)
ASD and typical controls, with = 10 individuals/group ABIDE I children and adults (i.e., PITT, NYU, USM) *n* = 211, ASD = 103, TD = 108 from three sites: (a) NYU Langone Medical Center (NYU, 35/51 ASD/controls); (b) University of Utah, School of Medicine (USM, 49/37 ASD/controls); (c) University of Pittsburg, School of Medicine (PITT, 19/20 ASD/controls) Replication data are from ABIDE II subsample, 103 individuals: (a) Trinity Centre for Health Sciences, Trinity College Dublin (TCD, 12/16 ASD/controls); (b) NYU Langone Medical Center (NYU, 25/18 ASD/controls); (c) Institut Pasteur/Robert Debré Hospital (IP, 11/21 ASD/controls)	Discovery Data ASD: 12.7–28.9 (mean: 20.8, *SD*: 8.1) TD: 12.1–26.3 (mean: 19.2, *SD*: 7.1)	Functional gradient analysis between core DMN regions and sensory regions (primary auditory, visual, and sensory-motor). Altered macroscale gradients and stepwise functional connectivity (SFC) and relationship with symptom severity.	Hong et al. (2019)
TD: 195, ASD: 170 (all ABIDE sites)	ASD: 8.22–22.92 (mean: 15.57, SD: 7.35) TD: 10.12–21.92 (mean: 16.02, *SD*: 5.90)	Dynamic functional network connectivity (dFNC) between 51 intrinsic connectivity network controls using independent component analysis and a sliding-window approach. A hard clustering state analysis and a fuzzy metastate analysis were conducted, respectively, for the exploration of local and global aberrant dynamic connectivity patterns in ASD. dFNC between thalamic and sensory networks in each functional state and group differences in four high-dimensional dynamic measures and relationship with symptom severity.	Fu et al. (2019)
TD: 26, ASD: 25 (all male adults)	TD: 18.1–39.4 (mean: 25.3, *SD*: 6.3) ASD: 18.4–50 (mean: 27.3, *SD*: 7.9)	Atypical intrinsic neural timescales estimated from sensory and core hub brain regions frontoparietal control, DMN, using autocorrelation function and related to underlying anatomical connectivity SC. Areas with shorter and longer timescales in the cortical hierarchy and relationship with symptom severity.	Watanabe et al. (2019)
ASD: 105, TD: 102 (all ABIDE sites)	TD: 7–12 (mean: 10.02, *SD*: 1.38) ASD: 7–12 (mean: 10.1, *SD*: 1.26)	Intra- and interhemispheric functional connectivity dynamics (FCD) mapping between core-peripheral brain regions and relationship with symptom severity.	Guo et al. (2020)

Accumulating evidence based on tracking dynamic changes in the autistic brain compared with neurotypical individuals suggests broader qualitative agreement across findings using open-access multisite neuroimaging data made available from ABIDE I and II (Di Martino et al., 2017; Di Martino et al., 2014). The availability of such large open-access multicentric datasets with unprecedented sample size and depth of phenotyping with balanced sex ratios allows for discovering fundamental neural mechanisms in spite of various sources of heterogeneity (Harlalka et al., 2019; Henry et al., 2018; Hong et al., 2019; Nomi, Bolt, et al., 2017; Rashid et al., 2018). The heterogeneity and site-related variability pose a severe caveat in the interpretation and replicability of functional connectivity findings, which is further described elsewhere in detail (King et al., 2019). Moreover, beyond sampling variability, the largest part of observed “dynamics” and time-varying FC configurations during rest is attributable to head motion (Laumann et al., 2017). Hence, while comparing the above metrics to establish between-group differences (specifically in children with autism) and relating these static and dynamic measures to symptom severity, one has to apply extreme caution as children with autism generally exhibit higher levels of head motion and thus pose an important caveat in interpretation.

However, several key challenges remain in the interpretation of functional connectivity–based findings; in general, resting-state BOLD correlations and brain dynamics may reflect processes concerned with maintenance of the long-term stability of the brain’s functional organization. Recent studies have further stratified samples at different stages of development (children, adolescents, and adults) to investigate how diagnosis by age interactions affect modularity, segregation, and integration in the functional brain networks. To address the stability and integrity of functional architecture, recent studies have further looked at the regional flexibility, cohesion, and disjointness in key core brain regions comprising the DMN, executive, and salience networks (Fu et al., 2019; Guo et al., 2017; Guo et al., 2020; Harlalka et al., 2018, 2019; He et al., 2018). These recent findings along with the previous findings using static brain network analysis quantifying normative network measures such as Rand index, density, and cohesiveness of regions of interest from ASD and TD suggest reduction in cohesion in somatosensory motor, auditory, subcortical, ventral attention, and memory retrieval regions in individuals with ASD compared with typically developing participants (Kenny et al., 2019). This was accounted for by globally reduced cohesion and density, but increased dispersion of the above networks (Kenny et al., 2019). Recent findings further suggest that there were significant differences in rich-club connectivity (among the hubs), which was generally increased in the ASD group. There are also hypervariant ASD connections in the dFCVar matrix (H. Chen et al., 2017; Guo et al., 2020; Harlalka et al., 2019). These hypervariant connections comprise inter- and intrahemispheric connectivity patterns (atypical long-range and short-range connection distributions). Recent evidence further suggests that a large majority of hypervariant connections in children and adults are long-range, while adolescents are seen to have a majority of short-range connections (Figure 2). In particular, it was reported that brain regions exhibiting reduced flexibility in ASD compared with TD are superior temporal gyrus (STG), putamen (PUT), amygdala (AMYG), cuneus (CUN), inferior occipital gyrus (IOG), left inferior parietal (IPL), and angular gyrus (ANG; Delmonte et al., 2013; Guo et al., 2017; Harlalka et al., 2018; Jao Keehn et al., 2019; Kenny et al., 2019). It was also reported that several regions that show effect of age include superior frontal orbital, amygdala (AMYG), cuneus (CUN), inferior occipital gyrus (IOG), left inferior parietal (IPL), angular gyrus (ANG), caudate nucleus (CAU), putamen (PUT), thalamus (THAL), dorsal SFG, and left superior temporal gyrus (STG; Harlalka et al., 2019). There were interesting group differences found between ASD and TD in pallidus gyrus (periphery region), which further showed a significant increase in flexibility in adults as compared with both adolescents and children, while the superior frontal orbital (periphery region) shows a significant increase of flexibility in adults compared with adolescents (Guo et al., 2020; Harlalka et al., 2019; Figure 3). In a recent study, autistic traits were accurately characterized using 774 children 6 to 10 years old based on the quantification of longer dwell times (less frequent state transitions in ASD compared with TD) and by global disconnection using dynamic functional connectivity analysis (Rashid et al., 2018). These findings suggest that globally atypical brain dynamics in ASD may be already present at the earliest stages of development and could be due to hub architecture being partly altered in ASD (Gollo et al., 2015; Keown et al., 2017). In summary, these findings support a core-periphery brain network dynamic model of reduced network integration (i.e., connectivity within networks) and differentiation (or segregation; based on connectivity outside network boundaries) in ASD (Guo et al., 2020; Harlalka et al., 2018, 2019; Keown et al., 2017; Preti & Van De Ville, 2019; Rashid et al., 2018).

Although reproducibility in findings based on static and dynamic functional connectivity methods described above is still a major hurdle in autism research (King et al., 2019), dynamic functional connectivity approaches are already uncovering atypical patterns of brain dynamics that distinguish autistic from neurotypical individuals in various developmental stages of relevance for sensory and cognitive behavioral deficits (H. Chen et al., 2017; Guo et al., 2020; Harlalka et al., 2019; He et al., 2018; Hong et al., 2019; Nomi, Bolt, et al., 2017; Nomi, Vij, et al., 2017; Rashid et al., 2018; Vidaurre et al., 2017; Watanabe & Rees, 2017; see Table 2 for a sample list of studies).

### Neural Substrates of Behavioral Variability in Autism: Prediction From Atypical Core-Periphery Timescales

In the past few years, understanding cortical hierarchical processing and timescales associated with core-periphery brain network dynamics has rapidly accelerated from primate to human studies in the quest to understand unifying principles of brain dynamics, hierarchical organization, and cognition (Bolton et al., 2020; Gollo et al., 2017; Gollo et al., 2015; Hasson et al., 2008; Hong et al., 2019; Khambhati et al., 2018; Kiebel et al., 2008; Liégeois et al., 2019; Nomi, Vij, et al., 2017; Preti & Van De Ville, 2019; Vidaurre et al., 2017; Watanabe et al., 2019). As such, the application of timescale separation and hierarchy of brain network dynamics in autism is relatively new, but the findings overlap significantly with findings based on other dynamical characterizations discussed here. Specifically, in autism, sensory stimuli often elicit delayed evoked responses in the auditory domain, and integration of multiple local stimuli into a global percept often requires a wider window of temporal binding (Robertson, Martin, Baker, & Baron-Cohen, 2012). In recent work using EEG- and fMRI-based neuroimaging evidence, it was found in healthy young individuals that the time differences of cross-modal perception may particularly tax multisensory processing and temporal dichotomy of dual-stream processing (streams for action versus stimulus awareness), in which local sensory stimuli must be integrated from two modalities (audio and visual, for example). In particular during dynamic perception, the sensory signals are built up and integrated over time (Kumar et al., 2016; Kumar, Dutta, Talwar, Roy, & Banerjee, 2012; Ray et al., 2020).

Hence, there is a growing concurrence among studies that in the autistic brain, the flexibility of brain dynamics is lost partly because of the distortion of timescales of integration of sensory input and routing and relaying of these sensory updates to higher order core brain regions for further processing in a hierarchical manner (Hong et al., 2019; Vidaurre et al., 2017; Watanabe & Rees, 2017; Watanabe et al., 2019).

A recent study using children 7–18 years old (not including 18 years) from ABIDE (Yao et al., 2016) found that those with ASD showed overall imbalance of strong and weakly connected brain regions, and decreased functional connectivity associated with DMN hub areas (precuneus/posterior cingulate gyrus with medial prefrontal gyrus). This study also found that compared with TD children, the strong connectivity over the peripheral sensory brain regions was maintained for a longer time (dwell time characterizing longer timescales of brain state occupation) between brain areas of children with ASD (exhibiting overstability), and ratios of weaker connectivity strength in brain states varied dramatically (exhibiting transient stability) in ASD (Yao et al., 2016). Hence, atypical connectivity strengths and states may be a macroscopical reflection of the excitatory/inhibitory imbalance at the cellular level (Yao et al., 2016).

Another recent study found reduced transitions between brain states in adults with autism using energy landscape analysis applied to seven well-established resting-state brain networks (Watanabe & Rees, 2017). Energy landscape and entropy analysis characterize atypical neural state transition probabilities between core DMN, executive control network (ECN), ventral attention network (VAN), dorsal attention network (DAN), and peripheral sensory networks and correlate with symptom severity. To quantify altered brain dynamics and state transitions in neurotypical individuals compared with ASD, the authors used an accurately fitted pairwise maximum entropy model (MEM) to estimate the energy landscape (Watanabe & Rees, 2017). They calculated so-called hypothetical energy values of all the possible brain activity patterns (in total 2^7^ patterns) among all the known seven resting-state networks (Watanabe & Rees, 2017). They examined hierarchal relationships between the 2^7^ energy values and systematically searched for dominant brain activity patterns that showed locally minimum energy values and that were more likely to be observed than similar activity patterns. They discovered that the TD and ASD groups had energy landscapes with similar hierarchal structures in the above networks. Their results suggest that direct transitions between major brain states are not different among the two groups but indirect transitions are. In individuals with autism, these atypical neural transitions are rare, which means that their brain dynamics in an energy landscape are more stable than in a neurotypical. Thus, their brain activity tends to occupy major brain states specifically in the visual, sensorimotor, and auditory networks for a sufficiently longer duration (Watanabe & Rees, 2017). While neurotypical individuals frequently transition between two major brain states via an intermediate state that has stability, high-functioning adults with ASD show fewer neural transitions because of an unstable intermediate state, and these infrequent transitions predict the severity of autism (Watanabe & Rees, 2017).

**Figure 4.**
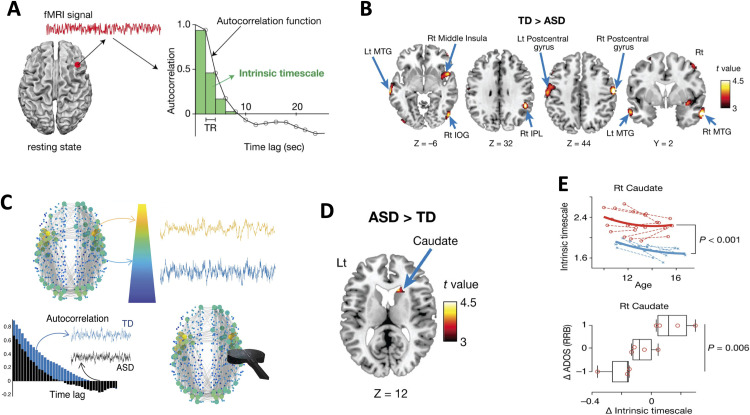
Atypical intrinsic neural timescale in autism. (A) Estimated neural timescale from fMRI BOLD signal, based on sum of autocorrelation function. (B) Intrinsic neural timescales are plotted in bilateral middle insula, pre- and postcentral gyrus (exhibiting shorter timescales). (C) Brain core regions located at the top of the hierarchy are shown in large (yellow) circles and have longer timescales. Brain regions located at the periphery are represented by small (blue) circles and have shorter timescales. Individuals with autism spectrum disorders (ASD, black) have different intrinsic timescales (quantified by the autocorrelation function) compared with typically developing individuals (TD, blue). A schematic displays that noninvasive brain stimulation (black coil) may be used to selectively modulate atypical brain regions to restore their intrinsic timescales. (D) Intrinsic neural timescales in the right caudate are longer in the ASD group compared with the TD group. (E) The intrinsic neural timescale in the right caudate is plotted as a function of age in TD (blue) and ASD (red) during adolescence and the correlation of intrinsic neural timescales with progression of RRB symptoms. Autocorrelation function, ACF; typically developing, TD; autism spectrum disorder, ASD. Figure adapted with permission from Watanabe et al. (2019).

Another recent finding introduces a very interesting and elegant approach to estimate an intrinsic neural timescale of an fMRI signal, based on the sum of autocorrelation function (ACF) values of the signals in the initial positive period of the ACF (Raut et al., 2020; Watanabe et al., 2019). The period is conceptually defined as an area under the ACF up to the time lag value (TR = 2 s) just before the one where the ACF becomes nonpositive for the first time as the time lag increases. The resulting sum when multiplied by the obtained area under the ACF by the repetition time (TR) is defined as the index of intrinsic timescale associated with a brain region of interest. Individuals with ASD had significantly shorter intrinsic timescales in bilateral postcentral gyri, right inferior parietal lobule (IPL), right middle insula, bilateral middle temporal gyri (MTG), and right inferior occipital gyrus (IOG), whereas the intrinsic timescale in the right caudate was significantly longer in the ASD group compared with the TD group (Figures 4A–E).The brain integrates incoming information over different timescales that are characteristic for different regions. Such a hierarchy of timescales also mirrors a hierarchy in brain structure (based on functional gradients in core-periphery interactions, see next section; Gollo, 2019; Hong et al., 2019; Raut et al., 2020; Watanabe et al., 2019). Brain regions located at the top of the hierarchy are represented as large (yellow) circles and have longer timescales. They are located at the core and have strong connections to other brain regions (Gollo, 2019; Hong et al., 2019; Raut et al., 2020; Watanabe et al., 2019). Brain regions located at the periphery are represented by small (blue) circles and have shorter timescales (Gollo, 2019; Raut et al., 2020; Watanabe et al., 2019). These differences correlate with the severity of symptoms of ASD. The intrinsic neural timescale in the right caudate was consistently longer in the ASD group during adolescence, which is interesting given that recent studies reported that loss of cohesiveness and flexibility in adolescence from right caudate regions could possibly indicate an impairment in motor coordination and restrictive behavior. In general, the overdevelopment of the intrinsic timescale was correlated with progression of RRB symptoms and replicates earlier findings that functional coordination in the brains of adults with ASD underpins overly stable neural dynamics, which supports both ASD core symptoms and cognitive abilities (Robertson & Baron-Cohen, 2017).

### Neural Substrates of Behavioral Variability in Autism: Prediction From Atypical Core-Periphery Functional Gradients

**Figure 5.**
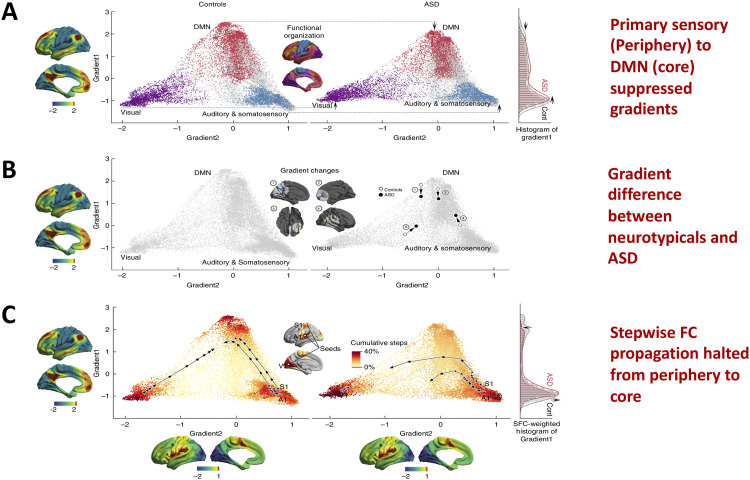
Atypical functional gradient and SFC from periphery to core brain regions in autism. (A) Scatterplot of the first two connectivity embedding gradients in controls and ASD. Gradient 1 (y-axis) runs from primary sensorimotor (dark turquoise) to transmodal DMN (sienna). Gradient 2 (x-axis) separates somatomotor and auditory cortex from visual cortex. Triangular scattered points are colored with respect to established functional communities. Histograms on right show the point density in ASD (light red) and controls (gray), suggesting overall compression of the first gradient in ASD. (B) Positional shifts of the four significant clusters from the surface-based analysis. (1) posterior cingulate cortex (PCC)/precuneus (PCU); (2) middle prefrontal cortex (mPFC); (3) occipito-temporal (OT); (4) posterior middle temporal gyrus (pMTG). (C) Stepwise functional connectivity (SFC) is estimated in the gradient space. Points are colored with respect to cumulative steps when simultaneously seeding from primary visual area (V1), somatosensory area (S1), and auditory area (A1). Trajectories (sampled every 20th step) illustrate the direct SFC from the primary sensory (periphery) seeds to transmodal DMN (core) in controls (left). ASD show an initially more rapid transition; however, trajectories deflect from a straight path and do not reach the DMN, even after 200 steps. Histograms on the right show point densities, weighted by the cumulative SFC. Figure adapted with permission from Hong et al. (2019).

Specific patterns of brain dynamics along the cortical hierarchy are associated with impairment of sensory and higher order cognitive processing in children, adolescents, and adults. Recent evidence suggests that mean variability of dFC between the attention and DMN networks is positively correlated with the Autism Diagnostic Observation Schedule (ADOS) scores (Douw, Wakeman, Tanaka, Liu, & Stufflebeam, 2016; Harlalka et al., 2019). This further suggests that intersubject variability is related to symptom severity and behavioral variability in task performance (Seghier & Price, 2018). Previous findings suggest that higher dFCVar values indicate better performance in task and poor performance in resting state (Douw et al., 2016). Similarly, higher variability in the functional connectivity strength of PCC to other DMN areas (within the same network) in the resting state is related to slower reaction times on a subsequent attention task (Lin et al., 2016; Liu et al., 2018). The hypervariance in ASD is an interesting observation and could potentially lead to a globally disconnected state between sensory and core brain areas (frontostriatal, fronto-occipital, DMN, SN, etc.). These results taken together indicate that there could be a relation between the atypical hypervariance in ASD which leads to an increase in ADOS scores and a decrease in cognitive performance. Recent studies also reported a significant number of hypervariable small-, medium-, and long-range connections in three groups (children, adolescents, adults) as shown in Figure 2. The long-range connections define the backbone of the functional network and often connect the hub regions to minimize wiring and energy costs (Deco et al., 2017; Gollo et al., 2015). In ASD, hypervariance in the long-range connections could cause instability in information transmission between hubs. Interestingly, for adolescents, recent studies found a higher number of hypervariable short-range connections (Harlalka et al., 2019). The hypervariance in short-range connections could indicate instability of local-module connectivity (Gollo et al., 2015). Further, several nodes, including orbitofrontal cortex and caudate, showed both hypervariability in connection strength and altered modular organization (flexibility) in ASD (Harlalka et al., 2019). A very recent finding further sought to resolve whether alterations in the macroscale hierarchy could provide a parsimonious explanation of the diverse symptoms (SA, RRB, and sociocognitive deficits) simultaneously (Hong et al., 2019). This study introduces a very elegant approach to quantify macroscale hierarchy by introducing a novel combination of connectome gradient and stepwise functional connectivity (SFC) analyses, which offer a complementary characterization of hierarchical brain anomalies in ASD. The gradient analysis and SFC estimation in ASD allow visualization of spatial trends in connectivity variations (as displayed in Figure 5) following the putative cortical hierarchy, while SFC is initiated from a priori selected sensory regions of interest to map stepwise connectivity transitions from peripheral nodes to core DMN nodes. Transmodal association cortices functionally shifted more toward peripheral sensory areas in ASD (Figure 5), a pattern that makes sensory input harder to ignore for participants with ASD. This may compromise higher order cognitive processing such as mentalization, communication, and speech processing by preventing the segregation of internally driven cognitive processes (Jasmin et al., 2019). These works are beginning to unravel the links between brain dynamics and flexible cognition and core symptoms such as RRBs (Bolton et al., 2020; Sahoo, Pathak, Deco, Banerjee, & Roy, 2020; Uddin, 2021).

To date, very few studies have explicitly explored the link between atypical flexibility, functional gradients, and timescales, and their relation to core sensory and higher order cognitive deficits. The hope is that with development of newer methods in the fields of brain dynamics and unsupervised and supervised machine learning, these types of early neuroimaging biomarkers may eventually pave the way to move from bench to bedside, and may predict idiosyncratic responses to interventions, as well as identify targeted treatment options for ASD.

## OUTSTANDING ISSUES AND FUTURE DIRECTIONS

### Neuroimaging of Individual Differences to Quantify Brain Dynamics of Atypical Core-Periphery Interactions

Based on the work we review here, it is quite apparent that understanding of large-scale brain network dynamics of core and periphery brain regions may provide critical insight into fundamental cognitive functions and flexibility associated with atypical neurodevelopment (Lynch et al., 2013; Gollo, 2019; Harlalka et al., 2019; Hong et al., 2019; Lin et al., 2016; Nomi & Uddin, 2015; Nomi, Vij, et al., 2017; Watanabe et al., 2019). For example, one of the key questions for future research is why individuals with autism display simultaneous impairment of sensory and higher order cognitive processing (altered local–global processing). We opine that this question is difficult to address without a proper dynamical framework to study neuroimaging data from individuals with autism. Past studies were largely unable to provide a detailed and satisfactory answer to reconcile these empirical observations (Lynch et al., 2013; Robertson, Martin, Baker, & Baron-Cohen, 2012). One may ask whether much of the higher order contextual cognitive deficits originate from a temporal processing problem. Altered temporal processing of sensory stimuli is observed in multiple early sensory modalities in autism (Ashwin, Ashwin, Rhydderch, Howells, & Baron-Cohen, 2009; Baron-Cohen et al., 2009; Robertson & Baron-Cohen, 2017).

Characterization of individual differences in brain state transitions (Rashid et al., 2018; Surampudi et al., 2019; Vidaurre et al., 2017; Watanabe & Rees, 2017) and core-periphery interactions in the brain across timescales and spatial hierarchies (sensory to transmodal cortex) may provide crucial insight to understand the dual impairments in autistic children, adolescents, and adults (Hahamy et al., 2015; He et al., 2018; Lin et al., 2016; Mottron et al., 2014; Pelphrey, Morris, & McCarthy, 2005; Watanabe & Rees, 2017; Watanabe et al., 2019). Interestingly, a recent approach going beyond identifying presence of community structure in brain networks but carrying out topological analysis of core-periphery structures has suggested the overall organization of whole-brain activity mapped at a single-participant level. This method, unlike most previous work, was able to successfully track both within- and between-task transitions from one task block to the next using topological analysis of core-periphery brain networks at the single-subject level (Saggar et al., 2018). Without the need to collapse neural data in space or time, one may gain useful information about the brain’s dynamical organization. Hence, combining topological analysis with atypical sensory timescales may present a promising avenue to investigate partial and full hub reorganization and contribute to the neural basis of individual differences and idiosyncrasy in ASD that remains an open question (Hahamy et al., 2015; Keown et al., 2017).

Recent work further suggests that autistic individuals with greater social impairment would require greater between-network integration to perform social tasks similarly to TD individuals (Jasmin et al., 2019). This compensatory neural strategy could be deployed in social situations outside the laboratory, too. It would be highly interesting to see whether core-periphery brain dynamics based on flexibility, functional gradients, and atypical timescales of sensory-motor areas may provide insights about whether autistic individuals using compensatory neural strategies show similar brain dynamics to neurotypical individuals.

Future studies are needed to systematically address several issues to make valuable inferences and predictions about the early onset of symptoms. First, there is limited generalizability and replicability across connectivity studies carried out in small and nonoverlapping samples, as seen from Table 2, and very little inclusion of females (Keown et al., 2017). Also, studies do not necessarily comprise individuals of varied socioeconomic status, who are largely underrepresented to date in neuroimaging research (Uddin, 2021).

### Consideration of Diagnosis by Age During Maturation of Core-Periphery Brain Networks

There are very few neuroimaging studies to date that have focused on age-wise stratifications and diagnosis by age while reporting brain connectivity and dynamic alterations observed in ASD. Additionally, it is completely unknown how the transitions from childhood to the chronological time that marks the beginning of adolescence influence the development of brain systems underlying core-periphery dynamics in autism and whether one can entirely rely on chronological timescales of development (Andreae, 2019; Baron-Cohen et al., 2009; Cheng et al., 2015; Cohen, 2018; Collignon et al., 2013; Di Martino et al., 2014; Uddin, 2021). Dissociable effects of hormonal changes and age on the adolescent brain have been well documented, suggesting that pubertal stage may be a better predictor of development of sensory and cognitive abilities and diversity of behavioral patterns than chronological age (Baum et al., 2017; Uddin, 2021). In this context, cognitive neuroscience work on ASD of children, adolescents, and adults needs to consider both hormonal changes during maturation as well as precise neuromodulatory effects on brain, behavior, and cognition (Liu et al., 2018; Reijmer et al., 2015; Shine, 2019). The findings of atypical network hierarchy, flexibility, and timescales of local sensory areas offer a novel and parsimonious account of the range of symptoms observed in ASD that encompasses multiple domains across sensory-motor, cognitive, and social-communicative functioning (Harlalka et al., 2019; Hong et al., 2019; Watanabe & Rees, 2017; Watanabe et al., 2019). In addition, the shorter intrinsic timescale found in the primary sensory/visual areas in autism was correlated with the overall severity of RRB in autism (Watanabe et al., 2019). In fact, the signal variability and the longer intrinsic timescale observed in the caudate nucleus were associated with the severity of RRBs in autistic individuals (Watanabe & Rees, 2017; Watanabe et al., 2019). In addition, a significant diagnosis by age interaction effect was observed in cohesion strength primarily in right caudate regions in autism but not in the left caudate (Figure 3). Taking together longer timescales and reduced cohesion strength particularly in the right caudate regions in ASD suggests that local processing in subcortical areas is significantly altered (Harlalka et al., 2019; Watanabe & Rees, 2017; Watanabe et al., 2019). Hence, future task-based neuroimaging studies need to focus on properly characterizing neural substrates of local subcortical regions and their role in atypical cognitive impairment. However, temporal properties of local neural signals have already been linked to local gray matter volumes (Watanabe et al., 2019). These crucial findings indicate the possibility that functional and structural properties in local brain areas and their strong coupling in sensory areas as opposed to divergence from tighter coupling in higher order transmodal areas (Baum et al., 2017; Murphy et al., 2018; Preti & Van De Ville, 2019; Reijmer et al., 2015; Vázquez-Rodríguez et al., 2019) could have a critical influence on higher order cognitive symptoms in autism (Cerliani et al., 2015; B. Chen et al., 2020; Kana et al., 2007). Furthermore, this possibility could be realized using gradient and SFC feature estimation techniques that could guide a supervised learning algorithm to predict symptom severity in individuals with ASD subtypes (Hong et al., 2019; Jao Keehn et al., 2019; Keown et al., 2017; Reiter et al., 2019; Uddin et al., 2013). These predictive features may for the first time allow us to understand the role of DMN, language areas in STG, and multisensory areas for processing dynamic stimuli in STS (transmodal cortex) in conjunction with peripheral sensory (auditory, visual, sensory-motor) and subcortical systems (thalamus, caudate, putamen) during maturation. This could resolve the puzzle of how both sensory and higher order aspects of the cortical hierarchy underpin ASD symptomatology.

## CONCLUSIONS

Core-periphery brain dynamics and atypical timescales of processing may facilitate optimal systems-level functioning in ASD. Studies of the neural mechanisms during various development stages are yet to be characterized comprehensively to fully understand atypical development and behavioral variability. To this end, one of the key challenges that remains is to understand how sensory deficits lead to higher order cognitive deficits in the domain of language, memory, attention, self-related processing, and executive functioning in ASD. This review provides an integrative view to explain simultaneity of sensory and cognitive deficits in autism, parsing through evidence from recent topological network-analysis-based approaches. More specifically, it highlights evidence that sensory and higher order cognitive deficits could be explained by atypical network flexibility, functional gradients, and timescale hierarchy in the cortex.

Moving forward, intrinsic timescales can be estimated using simple autocorrelations, which may be used to identify biomarkers and to improve our understanding of disease subtypes and treatment plans (Gollo, 2019). However, more research is necessary to fully comprehend and uncover the causes and implications of atypical intrinsic timescales. In individuals with autism, shorter timescales in unimodal sensory areas could relate to a heightened sensory perception and finer sensory discrimination, which is in line with excessive expectation of changes from the sensory environment. Moreover, longer timescales and reduced cohesiveness found in the caudate nucleus might also indicate a neural compensation strategy to deal with loaded sensory input due to heightened sensory perception and discrimination. In addition, not just sensory perception tasks but behaviorally and cognitively relevant social tasks can be carefully constructed to deal with varying cognitive demands in the service of attention and executive functions to allow atypical groups to carry out flexible task switching. The quantification of state-invariant core processing and state-dependent contextual processing can serve as a starting point for the characterization of a broader set of behavioral subtypes in autism. Translating these recent results into clinical practice will involve many practical challenges, but will be highly beneficial for furthering the neuroscience of autism.

## AUTHOR CONTRIBUTIONS

Dipanjan Roy: Conceptualization; Investigation; Methodology; Project administration; Visualization; Writing – original draft; Writing – review & editing. Lucina Q. Uddin: Conceptualization; Investigation; Methodology; Visualization; Writing – original draft; Writing – review & editing.

## FUNDING INFORMATION

Dipanjan Roy, Department of Biotechnology, Government of India, Award ID: BT/RLF/Re-entry/07/2014. Dipanjan Roy, Department of Science and Technology (DST) Ministry of Science and Technology, Government of India, Award ID: SR/CSRI/21/2016. Dipanjan Roy, BT/MED-III/NBRC/Flagship/Program/2019. Lucina Q. Uddin, National Institute of Mental Health, Award ID: R01MH107549. Lucina Q. Uddin, Canadian Institute for Advanced Research. Lucina Q. Uddin, Gabelli Senior Scholar Award, University of Miami.

## TECHNICAL TERMS

Network core: A set of densely connected brain regions that aggregate long-range connectivity and serve as a backbone for polymodal integration.
Network periphery: Brain networks primarily consisting of sensory and motor regions, with more locally clustered connectivity.
Flexibility: Flexibility is a network metric that characterizes the modular changes in each brain area throughout the scan period. However, this dynamic measure does not capture community affiliation.
Cohesion strength: In a network of brain areas, node strength is estimated as a cohesion matrix where the edge weights of the network denote the number of times a pair of nodes changes to the identical community affiliation.
Rich-club nodes: Strongly connected nodes in a network. If there is a core of such nodes with a high node degree that receives a very large proportion of connections and that are more densely interconnected among themselves than lower degree nodes in the network, they form a rich club. In other words, the high-degree nodes are densely connected hubs in the brain networks and form an exclusive club.
Transmodal cortex: This overarching system is thought to facilitate abstract, higher order cognitive functions by segregating information processing of the sensory environment from more self-generated and internally oriented cognition emerging in transmodal, integrative cortices.
Heteromodal association cortex: A region that receives input from multiple sensory or multimodal areas. These areas, including the prefrontal cortices, occipito-parieto-temporal junction, multisensory areas, and superior temporal cortex, are considered to play role in higher level cognition and context-dependent social processing.
Functional degeneracy: Well-known characteristics of a biological system whose elements that are structurally different to perform the same function or yield the same output.
Default mode network: Network of brain regions known to be active during the resting condition but that typically deactivate during the task condition. It is commonly related to self-referential thinking and daydreaming and represents intrinsic activity of the brain.
Executive control network: Large-scale control network consisting of flexible hubs that regulate distributed systems (e.g., visual, limbic, motor) according to current task goals.
Modularity: A graph theoretic measure for the strength of division of a network into modules (or communities). Networks with high modularity have dense connections between the nodes within modules, but sparse connections between nodes in different modules.
Node disjointness: Node disjointness defines the fraction of time a node changes its community affiliation over time, independent of the other nodes.
Rand index: A network measure reflecting how similar network organization was to a normative set of networks. This measure compares similarity between two clustering assignments and can be used for comparison against a normative set of labels.
Intrinsic timescales: A neuroimaging analysis technique that allows estimation of an intrinsic neural timescale of an fMRI signal as the sum of autocorrelation function (ACF) values of the signals in the initial positive period of the ACF. The period is defined as the area under the ACF up to the time lag value just before the one where the ACF becomes nonpositive for the first time as the time lag increases. Subsequently, multiplying the obtained area under the ACF by the repetition time (TR) formally defines the index for the intrinsic timescale.
Connectome gradient: A graph signal processing technique that estimates a low-dimensional embedding from a high-dimensional functional connectivity matrix derived from all brain regions of interest. In this space, cortical vertices that are strongly interconnected by either many connections or a few very strong connections are closer together, whereas vertices with only little or no interconnectivity are farther apart, giving a gradient of information flow in the anatomical connectivity space.
Stepwise functional connectivity: Stepwise functional connectivity (SFC) analysis estimates Pearson correlation strength between seed (e.g., sensory-motor) and target (DMN areas) brain regions as a sequence of steps in connectivity space. Usually, for information reaching from sensory to higher order cortical areas, seed-to-target functional connectivity estimation in principle should converge in fewer steps (e.g., 20–30 steps).

## References

[bib1] Abbott, A. E., Linke, A. C., Nair, A., Jahedi, A., Alba, L. A., Keown, C. L., … Müller, R. A. (2018). Repetitive behaviors in autism are linked to imbalance of corticostriatal connectivity: A functional connectivity MRI study. Social Cognitive and Affective Neuroscience, 13(1), 32–42. **DOI:** https://doi.org/10.1093/scan/nsx129, **PMID:** 29177509, **PMCID:** PMC5793718 29177509 10.1093/scan/nsx129 PMC5793718

[bib2] Alaerts, K., Nayar, K., Kelly, C., Raithel, J., Milham, M. P., & Di Martino, A. (2015). Age-related changes in intrinsic function of the superior temporal sulcus in autism spectrum disorders. Social Cognitive and Affective Neuroscience, 10(10), 1413–1423. **DOI:** https://doi.org/10.1093/scan/nsv029, **PMID:** 25809403, **PMCID:** PMC4590540 25809403 10.1093/scan/nsv029 PMC4590540

[bib3] Alaerts, K., Swinnen, S. P., & Wenderoth, N. (2016). Sex differences in autism: A resting-state fMRI investigation of functional brain connectivity in males and females. Social Cognitive and Affective Neuroscience, 11(6), 1002–1016. **DOI:** https://doi.org/10.1093/scan/nsw027, **PMID:** 26989195, **PMCID:** PMC4884321 26989195 10.1093/scan/nsw027 PMC4884321

[bib4] Alaerts, K., Woolley, D. G., Steyaert, J., Di Martino, A., Swinnen, S. P., & Wenderoth, N. (2014). Underconnectivity of the superior temporal sulcus predicts emotion recognition deficits in autism. Social Cognitive and Affective Neuroscience, 9(10), 1589–1600. **DOI:** https://doi.org/10.1093/scan/nst156, **PMID:** 24078018, **PMCID:** PMC4187281 24078018 10.1093/scan/nst156 PMC4187281

[bib5] Anderson, J. S., Druzgal, T. J., Froehlich, A., DuBray, M. B., Lange, N., Alexander, A. L., … Bigler, E. D. (2011). Decreased interhemispheric functional connectivity in autism. Cerebral Cortex, 21(5), 1134–1146. **DOI:** https://doi.org/10.1093/cercor/bhq190, **PMID:** 20943668, **PMCID:** PMC3077433 20943668 10.1093/cercor/bhq190 PMC3077433

[bib6] Andreae, L. C. (2019). Brain development in autism: Timing is everything. Science Translational Medicine, 11(476). 10.1126/scitranslmed.aaw5314

[bib7] Ashwin, E., Ashwin, C., Rhydderch, D., Howells, J., & Baron-Cohen, S. (2009). Eagle-eyed visual acuity: An experimental investigation of enhanced perception in autism. Biological Psychiatry, 65(1), 17–21. **DOI:** https://doi.org/10.1016/j.biopsych.2008.06.012, **PMID:** 18649873 18649873 10.1016/j.biopsych.2008.06.012

[bib8] Atasoy, S., Donnelly, I., & Pearson, J. (2016). Human brain networks function in connectome-specific harmonic waves. Nature Communications, 7, 10340. **DOI:** https://doi.org/10.1038/ncomms10340, **PMID:** 26792267, **PMCID:** PMC4735826 10.1038/ncomms10340 PMC4735826 26792267

[bib9] Baron-Cohen, S., Ashwin, E., Ashwin, C., Tavassoli, T., & Chakrabarti, B. (2009). Talent in autism: Hyper-systemizing, hyper-attention to detail and sensory hypersensitivity. Philosophical Transactions of the Royal Society B: Biological Sciences, 364(1522), 1377–1383. **DOI:** https://doi.org/10.1098/rstb.2008.0337, **PMID:** 19528020, **PMCID:** PMC2677592 10.1098/rstb.2008.0337 PMC2677592 19528020

[bib10] Baum, G. L., Ciric, R., Roalf, D. R., Betzel, R. F., Moore, T. M., Shinohara, R. T., … Cook, P. A. (2017). Modular segregation of structural brain networks supports the development of executive function in youth. Current Biology, 27(11), 1561–1572. **DOI:** https://doi.org/10.1016/j.cub.2017.04.051, **PMID:** 28552358, **PMCID:** PMC5491213 28552358 10.1016/j.cub.2017.04.051 PMC5491213

[bib11] Blakemore, S. J., Burnett, S., & Dahl, R. E. (2010). The role of puberty in the developing adolescent brain. Human Brain Mapping, 31(6), 926–933. **DOI:** https://doi.org/10.1002/hbm.21052, **PMID:** 20496383, **PMCID:** PMC3410522 20496383 10.1002/hbm.21052 PMC3410522

[bib12] Bolton, T. A., Morgenroth, E., Preti, M. G., & Van De Ville, D. (2020). Tapping into multi-faceted human behavior and psychopathology using fMRI brain dynamics. Trends in Neurosciences, 43(9), 667–680. **DOI:** https://doi.org/10.1016/j.tins.2020.06.005, **PMID:** 32682563 32682563 10.1016/j.tins.2020.06.005

[bib13] Cerliani, L., Mennes, M., Thomas, R. M., Di Martino, A., Thioux, M., & Keysers, C. (2015). Increased functional connectivity between subcortical and cortical resting-state networks in autism spectrum disorder. JAMA Psychiatry, 72(8), 767–777. **DOI:** https://doi.org/10.1001/jamapsychiatry.2015.0101, **PMID:** 26061743, **PMCID:** PMC5008437 26061743 10.1001/jamapsychiatry.2015.0101 PMC5008437

[bib14] Chang, C., & Glover, G. H. (2010). Time-frequency dynamics of resting-state brain connectivity measured with fMRI. NeuroImage, 50(1), 81–98. **DOI:** https://doi.org/10.1016/j.neuroimage.2009.12.011, **PMID:** 20006716, **PMCID:** PMC2827259 20006716 10.1016/j.neuroimage.2009.12.011 PMC2827259

[bib15] Chaudhuri, R., Knoblauch, K., Gariel, M. A., Kennedy, H., & Wang, X. J. (2015). A large-scale circuit mechanism for hierarchical dynamical processing in the primate cortex. Neuron, 88(2), 419–431. **DOI:** https://doi.org/10.1016/j.neuron.2015.09.008, **PMID:** 26439530, **PMCID:** PMC4630024 26439530 10.1016/j.neuron.2015.09.008 PMC4630024

[bib16] Chen, B., Linke, A., Olson, L., Ibarra, C., Reynolds, S., Müller, R. A., … Fishman, I. (2020). Greater functional connectivity between sensory networks is related to symptom severity in toddlers with autism spectrum disorder. Journal of Child Psychology and Psychiatry. **DOI:** https://doi.org/10.1111/jcpp.13268, **PMID:** PMC7688487 10.1111/jcpp.13268 PMC7688487 32452051

[bib17] Chen, H., Nomi, J. S., Uddin, L. Q., Duan, X., & Chen, H. (2017). Intrinsic functional connectivity variance and state-specific under-connectivity in autism. Human Brain Mapping, 38(11), 5740–5755. **DOI:** https://doi.org/10.1002/hbm.23764, **PMID:** 28792117, **PMCID:** PMC5783325 28792117 10.1002/hbm.23764 PMC5783325

[bib18] Cheng, W., Rolls, E. T., Gu, H., Zhang, J., & Feng, J. (2015). Autism: Reduced connectivity between cortical areas involved in face expression, theory of mind, and the sense of self. Brain, 138(5), 1382–1393. **DOI:** https://doi.org/10.1093/brain/awv051, **PMID:** 25795704, **PMCID:** PMC4407191 25795704 10.1093/brain/awv051 PMC4407191

[bib19] Ciarrusta, J., O’Muircheartaigh, J., Dimitrova, R., Batalle, D., Cordero-Grande, L., Price, A., … Javed, A. (2019). Social brain functional maturation in newborn infants with and without a family history of autism spectrum disorder. JAMA Network Open, 2(4), e191868. **DOI:** https://doi.org/10.1001/jamanetworkopen.2019.1868, **PMID:** 30951164, **PMCID:** PMC6450332 30951164 10.1001/jamanetworkopen.2019.1868 PMC6450332

[bib20] Cohen, J. R. (2018). The behavioral and cognitive relevance of time-varying, dynamic changes in functional connectivity. NeuroImage, 180, 515–525. **DOI:** https://doi.org/10.1016/j.neuroimage.2017.09.036, **PMID:** 28942061, **PMCID:** PMC6056319 28942061 10.1016/j.neuroimage.2017.09.036 PMC6056319

[bib21] Collignon, O., Charbonneau, G., Peters, F., Nassim, M., Lassonde, M., Lepore, F., … Bertone, A. (2013). Reduced multisensory facilitation in persons with autism. Cortex, 49(6), 1704–1710. **DOI:** https://doi.org/10.1016/j.cortex.2012.06.001, **PMID:** 22818902 22818902 10.1016/j.cortex.2012.06.001

[bib22] Courchesne, E., Campbell, K., & Solso, S. (2011). Brain growth across the life span in autism: Age-specific changes in anatomical pathology. Brain Research, 1380, 138–145. **DOI:** https://doi.org/10.1016/j.brainres.2010.09.101, **PMID:** 20920490, **PMCID:** PMC4500507 20920490 10.1016/j.brainres.2010.09.101 PMC4500507

[bib23] Dajani, D. R., Odriozola, P., Winters, M., Voorhies, W., Marcano, S., Baez, A., … Uddin, L. Q. (2020). Measuring cognitive flexibility with the flexible item selection task: From fMRI adaptation to individual connectome mapping. Journal of Cognitive Neuroscience, 32(6), 1026–1045. **DOI:** https://doi.org/10.1162/jocn_a_01536, **PMID:** 32013686 32013686 10.1162/jocn_a_01536

[bib24] Deco, G., Kringelbach, M. L., Jirsa, V. K., & Ritter, P. (2017). The dynamics of resting fluctuations in the brain: Metastability and its dynamical cortical core. Scientific Reports, 7(1), 1–14. **DOI:** https://doi.org/10.1038/s41598-017-03073-5, **PMID:** 28596608, **PMCID:** PMC5465179 28596608 10.1038/s41598-017-03073-5 PMC5465179

[bib25] Delmonte, S., Gallagher, L., O’Hanlon, E., McGrath, J., & Balsters, J. H. (2013). Functional and structural connectivity of frontostriatal circuitry in autism spectrum disorder. Frontiers in Human Neuroscience, 7, 430. **DOI:** https://doi.org/10.3389/fnhum.2013.00430, **PMID:** 23964221, **PMCID:** PMC3734372 23964221 10.3389/fnhum.2013.00430 PMC3734372

[bib26] Di Martino, A., O’Connor, D., Chen, B., Alaerts, K., Anderson, J. S., Assaf, M., … Blanken, L. M. (2017). Enhancing studies of the connectome in autism using the Autism Brain Imaging Data Exchange II. Scientific Data, 4(1), 1–15. **DOI:** https://doi.org/10.1038/sdata.2017.10, **PMID:** 28291247, **PMCID:** PMC5349246 10.1038/sdata.2017.10 PMC5349246 28291247

[bib27] Di Martino, A., Yan, C. G., Li, Q., Denio, E., Castellanos, F. X., Alaerts, K., … Deen, B. (2014). The Autism Brain Imaging Data Exchange: Towards a large-scale evaluation of the intrinsic brain architecture in autism. Molecular Psychiatry, 19(6), 659–667. **DOI:** https://doi.org/10.1038/mp.2013.78, **PMID:** 23774715, **PMCID:** PMC4162310 23774715 10.1038/mp.2013.78 PMC4162310

[bib28] Douw, L., Wakeman, D. G., Tanaka, N., Liu, H., & Stufflebeam, S. M. (2016). State-dependent variability of dynamic functional connectivity between frontoparietal and default networks relates to cognitive flexibility. Neuroscience, 339, 12–21. **DOI:** https://doi.org/10.1016/j.neuroscience.2016.09.034, **PMID:** 27687802, **PMCID:** PMC5635855 27687802 10.1016/j.neuroscience.2016.09.034 PMC5635855

[bib29] Foxe, J. J., Molholm, S., Del Bene, V. A., Frey, H. P., Russo, N. N., Blanco, D., … Ross, L. A. (2015). Severe multisensory speech integration deficits in high-functioning school-aged children with autism spectrum disorder (ASD) and their resolution during early adolescence. Cerebral Cortex, 25(2), 298–312. **DOI:** https://doi.org/10.1093/cercor/bht213, **PMID:** 23985136, **PMCID:** PMC4303800 23985136 10.1093/cercor/bht213 PMC4303800

[bib30] Friston, K. (2009). The free-energy principle: A rough guide to the brain? Trends in Cognitive Sciences, 13(7), 293–301. **DOI:** https://doi.org/10.1016/j.tics.2009.04.005, **PMID:** 19559644 19559644 10.1016/j.tics.2009.04.005

[bib31] Fu, Z., Tu, Y., Di, X., Du, Y., Sui, J., Biswal, B. B., … Calhoun, V. D. (2019). Transient increased thalamic-sensory connectivity and decreased whole-brain dynamism in autism. NeuroImage, 190, 191–204. **DOI:** https://doi.org/10.1016/j.neuroimage.2018.06.003, **PMID:** 29883735, **PMCID:** PMC6281849 29883735 10.1016/j.neuroimage.2018.06.003 PMC6281849

[bib32] Gollo, L. L. (2019). Computational psychiatry: Exploring atypical timescales in the brain. eLife, 8, e45089. **DOI:** https://doi.org/10.7554/eLife.45089, **PMID:** 30717825, **PMCID:** PMC6363382 30717825 10.7554/eLife.45089 PMC6363382

[bib33] Gollo, L. L., Roberts, J. A., & Cocchi, L. (2017). Mapping how local perturbations influence systems-level brain dynamics. NeuroImage, 160, 97–112. **DOI:** https://doi.org/10.1016/j.neuroimage.2017.01.057, **PMID:** 28126550 28126550 10.1016/j.neuroimage.2017.01.057

[bib34] Gollo, L. L., Zalesky, A., Hutchison, R. M., van den Heuvel, M., & Breakspear, M. (2015). Dwelling quietly in the rich club: Brain network determinants of slow cortical fluctuations. Philosophical Transactions of the Royal Society B: Biological Sciences, 370(1668), 20140165. **DOI:** https://doi.org/10.1098/rstb.2014.0165, **PMID:** 25823864, **PMCID:** PMC4387508 10.1098/rstb.2014.0165 PMC4387508 25823864

[bib35] Guo, X., Chen, H., Long, Z., Duan, X., Zhang, Y., & Chen, H. (2017). Atypical developmental trajectory of local spontaneous brain activity in autism spectrum disorder. Scientific Reports, 7(1), 1–10. **DOI:** https://doi.org/10.1038/srep39822, **PMID:** 28057930, **PMCID:** PMC5216408 28057930 10.1038/srep39822 PMC5216408

[bib36] Guo, X., Duan, X., Chen, H., He, C., Xiao, J., Han, S., … Chen, H. (2020). Altered inter-and intrahemispheric functional connectivity dynamics in autistic children. Human Brain Mapping, 41(2), 419–428. **DOI:** https://doi.org/10.1002/hbm.24812, **PMID:** 31600014, **PMCID:** PMC7268059 31600014 10.1002/hbm.24812 PMC7268059

[bib37] Guo, X., Duan, X., Suckling, J., Chen, H., Liao, W., Cui, Q., & Chen, H. (2019). Partially impaired functional connectivity states between right anterior insula and default mode network in autism spectrum disorder. Human Brain Mapping, 40(4), 1264–1275. **DOI:** https://doi.org/10.1002/hbm.24447, **PMID:** 30367744, **PMCID:** PMC6865537 30367744 10.1002/hbm.24447 PMC6865537

[bib38] Griffa, A., & van den Heuvel, M. P. (2018). Rich-club neurocircuitry: Function, evolution, and vulnerability. Dialogues in Clinical Neuroscience, 20(2), 121. **DOI:** https://doi.org/10.31887/DCNS.2018.20.2/agriffa, **PMID:** 30250389, **PMCID:** PMC6136122 30250389 10.31887/DCNS.2018.20.2/agriffa PMC6136122

[bib39] Hahamy, A., Behrmann, M., & Malach, R. (2015). The idiosyncratic brain: Distortion of spontaneous connectivity patterns in autism spectrum disorder. Nature Neuroscience, 18(2), 302. **DOI:** https://doi.org/10.1038/nn.3919, **PMID:** 25599222 25599222 10.1038/nn.3919

[bib40] Harlalka, V., Bapi, R. S., Vinod, P. K., & Roy, D. (2018). Age, disease, and their interaction effects on intrinsic connectivity of children and adolescents in autism spectrum disorder using functional connectomics. Brain Connectivity, 8(7), 407–419. **DOI:** https://doi.org/10.1089/brain.2018.0616, **PMID:** 30009617 30009617 10.1089/brain.2018.0616

[bib41] Harlalka, V., Bapi, R. S., Vinod, P. K., & Roy, D. (2019). Atypical flexibility in dynamic functional connectivity quantifies the severity in autism spectrum disorder. Frontiers in Human Neuroscience, 13, 6. **DOI:** https://doi.org/10.3389/fnhum.2019.00006, **PMID:** 30774589, **PMCID:** PMC6367662 30774589 10.3389/fnhum.2019.00006 PMC6367662

[bib42] Hasson, U., Yang, E., Vallines, I., Heeger, D. J., & Rubin, N. (2008). A hierarchy of temporal receptive windows in human cortex. Journal of Neuroscience, 28(10), 2539–2550. **DOI:** https://doi.org/10.1523/JNEUROSCI.5487-07.2008, **PMID:** 18322098, **PMCID:** PMC2556707 18322098 10.1523/JNEUROSCI.5487-07.2008 PMC2556707

[bib43] He, C., Chen, Y., Jian, T., Chen, H., Guo, X., Wang, J., … Duan, X. (2018). Dynamic functional connectivity analysis reveals decreased variability of the default-mode network in developing autistic brain. Autism Research, 11(11), 1479–1493. **DOI:** https://doi.org/10.1002/aur.2020, **PMID:** 30270547 30270547 10.1002/aur.2020

[bib44] Henry, T. R., Dichter, G. S., & Gates, K. (2018). Age and gender effects on intrinsic connectivity in autism using functional integration and segregation. Biological Psychiatry: Cognitive Neuroscience and Neuroimaging, 3(5), 414–422. **DOI:** https://doi.org/10.1016/j.bpsc.2017.10.006, **PMID:** 29735152 29735152 10.1016/j.bpsc.2017.10.006

[bib45] Hilgetag, C. C., & Goulas, A. (2020). ‘Hierarchy’ in the organization of brain networks. Philosophical Transactions of the Royal Society B, 375(1796), 20190319. **DOI:** https://doi.org/10.1098/rstb.2019.0319, **PMID:** 32089116, **PMCID:** PMC7061955 10.1098/rstb.2019.0319 PMC7061955 32089116

[bib46] Hong, S-J., Vos De Wael, R., Bethlehem, R. A. I., Lariviere, S., Paquola, C., Valk, S. L., … Bernhardt, B. C. (2019). Atypical functional connectome hierarchy in autism. Nature Communications, 10(1), 1–13. **DOI:** https://doi.org/10.1038/s41467-019-08944-1, **PMID:** 30833582, **PMCID:** PMC6399265 10.1038/s41467-019-08944-1 PMC6399265 30833582

[bib47] Huang, H., Shu, N., Mishra, V., Jeon, T., Chalak, L., Wang, Z. J., … Dong, Q. (2015). Development of human brain structural networks through infancy and childhood. Cerebral Cortex, 25(5), 1389–1404. **DOI:** https://doi.org/10.1093/cercor/bht335, **PMID:** 24335033, **PMCID:** PMC4397575 24335033 10.1093/cercor/bht335 PMC4397575

[bib48] Jao Keehn, R. J., Nair, S., Pueschel, E. B., Linke, A. C., Fishman, I., & Müller, R. A. (2019). Atypical local and distal patterns of occipito-frontal functional connectivity are related to symptom severity in autism. Cerebral Cortex, 29(8), 3319–3330. **DOI:** https://doi.org/10.1093/cercor/bhy201, **PMID:** 30137241, **PMCID:** PMC7342606 30137241 10.1093/cercor/bhy201 PMC7342606

[bib49] Jao Keehn, R. J., Sanchez, S. S., Stewart, C. R., Zhao, W., Grenesko-Stevens, E. L., Keehn, B., & Müller, R. A. (2017). Impaired downregulation of visual cortex during auditory processing is associated with autism symptomatology in children and adolescents with autism spectrum disorder. Autism Research, 10(1), 130–143. **DOI:** https://doi.org/10.1002/aur.1636, **PMID:** 27205875, **PMCID:** PMC5892834 27205875 10.1002/aur.1636 PMC5892834

[bib50] Jasmin, K., Gotts, S. J., Xu, Y., Liu, S., Riddell, C. D., Ingeholm, J. E., … Martin, A. (2019). Overt social interaction and resting state in young adult males with autism: Core and contextual neural features. Brain, 142(3), 808–822. **DOI:** https://doi.org/10.1093/brain/awz003, **PMID:** 30698656, **PMCID:** PMC6391610 30698656 10.1093/brain/awz003 PMC6391610

[bib51] Kana, R. K., Keller, T. A., Minshew, N. J., & Just, M. A. (2007). Inhibitory control in high-functioning autism: Decreased activation and underconnectivity in inhibition networks. Biological Psychiatry, 62(3), 198–206. **DOI:** https://doi.org/10.1016/j.biopsych.2006.08.004, **PMID:** 17137558, **PMCID:** PMC4492460 17137558 10.1016/j.biopsych.2006.08.004 PMC4492460

[bib52] Kenny, L., Cribb, S. J., & Pellicano, E. (2019). Childhood executive function predicts later autistic features and adaptive behavior in young autistic people: A 12-year prospective study. Journal of Abnormal Child Psychology, 47(6), 1089–1099. **DOI:** https://doi.org/10.1007/s10802-018-0493-8, **PMID:** 30421376 30421376 10.1007/s10802-018-0493-8

[bib53] Keown, C. L., Datko, M. C., Chen, C. P., Maximo, J. O., Jahedi, A., & Müller, R. A. (2017). Network organization is globally atypical in autism: A graph theory study of intrinsic functional connectivity. Biological Psychiatry: Cognitive Neuroscience and Neuroimaging, 2(1), 66–75. **DOI:** https://doi.org/10.1016/j.bpsc.2016.07.008, **PMID:** 28944305, **PMCID:** PMC5607014 28944305 10.1016/j.bpsc.2016.07.008 PMC5607014

[bib54] Khambhati, A. N., Medaglia, J. D., Karuza, E. A., Thompson-Schill, S. L., & Bassett, D. S. (2018). Subgraphs of functional brain networks identify dynamical constraints of cognitive control. PLoS Computational Biology, 14(7), e1006234. **DOI:** https://doi.org/10.1371/journal.pcbi.1006234, **PMID:** 29979673, **PMCID:** PMC6056061 29979673 10.1371/journal.pcbi.1006234 PMC6056061

[bib55] Kiebel, S. J., Daunizeau, J., & Friston, K. J. (2008). A hierarchy of timescales and the brain. PLoS Computational Biology, 4(11), e1000209. **DOI:** https://doi.org/10.1371/journal.pcbi.1000209, **PMID:** 19008936, **PMCID:** PMC2568860 19008936 10.1371/journal.pcbi.1000209 PMC2568860

[bib56] King, J. B., Prigge, M. B., King, C. K., Morgan, J., Weathersby, F., Fox, J. C., … Bigler, E. D. (2019). Generalizability and reproducibility of functional connectivity in autism. Molecular Autism, 10(1), 27. **DOI:** https://doi.org/10.1186/s13229-019-0273-5, **PMID:** 31285817, **PMCID:** PMC6591952 31285817 10.1186/s13229-019-0273-5 PMC6591952

[bib57] Kumar, V. G., Halder, T., Jaiswal, A. K., Mukherjee, A., Roy, D., & Banerjee, A. (2016). Large scale functional brain networks underlying temporal integration of audio-visual speech perception: An EEG study. Frontiers in Psychology, 7, 1558. **DOI:** https://doi.org/10.3389/fpsyg.2016.01558, **PMID:** 27790169, **PMCID:** PMC5062921 27790169 10.3389/fpsyg.2016.01558 PMC5062921

[bib58] Kumar, V. G., Dutta, S., Talwar, S., Roy, D., & Banerjee, A. (2020). Biophysical mechanisms governing large-scale brain network dynamics underlying individual-specific variability of perception. European Journal of Neuroscience, 52(7), 3746–3762. **DOI:** https://doi.org/10.1111/ejn.14747, **PMID:** 32304122 10.1111/ejn.14747 32304122

[bib59] Lai, M. C., Lerch, J. P., Floris, D. L., Ruigrok, A. N., Pohl, A., Lombardo, M. V., & Baron-Cohen, S. (2017). Imaging sex/gender and autism in the brain: Etiological implications. Journal of Neuroscience Research, 95(1–2), 380–397. **DOI:** https://doi.org/10.1002/jnr.23948, **PMID:** 27870420 27870420 10.1002/jnr.23948

[bib60] Laumann, T. O., Snyder, A. Z., Mitra, A., Gordon, E. M., Gratton, C., Adeyemo, B., … McCarthy, J. E. (2017). On the stability of BOLD fMRI correlations. Cerebral Cortex, 27(10), 4719–4732. 27591147 10.1093/cercor/bhw265 PMC6248456

[bib61] Lawson, R. A., Papadakis, A. A., Higginson, C. I., Barnett, J. E., Wills, M. C., Strang, J. F., … Kenworthy, L. (2015). Everyday executive function impairments predict comorbid psychopathology in autism spectrum and attention deficit hyperactivity disorders. Neuropsychology, 29(3), 445. **DOI:** https://doi.org/10.1037/neu0000145, **PMID:** 25313979 25313979 10.1037/neu0000145

[bib62] Liégeois, R., Li, J., Kong, R., Orban, C., Van De Ville, D., Ge, T., … Yeo, B. T. (2019). Resting brain dynamics at different timescales capture distinct aspects of human behavior. Nature Communications, 10(1), 1–9. **DOI:** https://doi.org/10.1038/s41467-019-10317-7, **PMID:** 31127095, **PMCID:** PMC6534566 10.1038/s41467-019-10317-7 PMC6534566 31127095

[bib63] Lin, P., Yang, Y., Jovicich, J., De Pisapia, N., Wang, X., Zuo, C. S., & Levitt, J. J. (2016). Static and dynamic posterior cingulate cortex nodal topology of default mode network predicts attention task performance. Brain Imaging and Behavior, 10, 212–225. **DOI:** https://doi.org/10.1007/s11682-015-9384-6, **PMID:** 25904156 25904156 10.1007/s11682-015-9384-6

[bib64] Liu, J., Liao, X., Xia, M., & He, Y. (2018). Chronnectome fingerprinting: Identifying individuals and predicting higher cognitive functions using dynamic brain connectivity patterns. Human Brain Mapping, 39(2), 902–915. **DOI:** https://doi.org/10.1002/hbm.23890, **PMID:** 29143409, **PMCID:** PMC6866558 29143409 10.1002/hbm.23890 PMC6866558

[bib65] London, E. B. (2018). Neuromodulation and a reconceptualization of autism spectrum disorders: Using the locus coeruleus functioning as an exemplar. Frontiers in Neurology, 9, 1120. **DOI:** https://doi.org/10.3389/fneur.2018.01120, **PMID:** 30619071, **PMCID:** PMC6305710 30619071 10.3389/fneur.2018.01120 PMC6305710

[bib66] Lynch, C. J., Uddin, L. Q., Supekar, K., Khouzam, A., Phillips, J., & Menon, V. (2013). Default mode network in childhood autism: posteromedial cortex heterogeneity and relationship with social deficits. Biological Psychiatry, 74(3), 212–219. **DOI:** https://doi.org/10.1016/j.biopsych.2012.12.013, **PMID:** 23375976, **PMCID:** PMC3710546 23375976 10.1016/j.biopsych.2012.12.013 PMC3710546

[bib67] Manning, C., Tibber, M. S., Charman, T., Dakin, S. C., & Pellicano, E. (2015). Enhanced integration of motion information in children with autism. Journal of Neuroscience, 35(18), 6979–6986. **DOI:** https://doi.org/10.1523/JNEUROSCI.4645-14.2015, **PMID:** 25948250, **PMCID:** PMC4420775 25948250 10.1523/JNEUROSCI.4645-14.2015 PMC4420775

[bib68] Mash, L. E., Reiter, M. A., Linke, A. C., Townsend, J., & Müller, R. A. (2018). Multimodal approaches to functional connectivity in autism spectrum disorders: An integrative perspective. Developmental Neurobiology, 78(5), 456–473. **DOI:** https://https://doi.org/10.1002/dneu.22570, **PMID:** 29266810, **PMCID:** PMC5897150 29266810 10.1002/dneu.22570 PMC5897150

[bib69] McKinnon, C. J., Eggebrecht, A. T., Todorov, A., Wolff, J. J., Elison, J. T., Adams, C. M., … McKinstry, R. C. (2019). Restricted and repetitive behavior and brain functional connectivity in infants at risk for developing autism spectrum disorder. Biological Psychiatry: Cognitive Neuroscience and Neuroimaging, 4(1), 50–61. **DOI:** https://doi.org/10.1016/j.bpsc.2018.09.008, **PMID:** 30446435, **PMCID:** PMC6557405 30446435 10.1016/j.bpsc.2018.09.008 PMC6557405

[bib70] Mottron, L., Belleville, S., Rouleau, G. A., & Collignon, O. (2014). Linking neocortical, cognitive, and genetic variability in autism with alterations of brain plasticity: The Trigger-Threshold-Target model. Neuroscience and Biobehavioral Reviews, 47, 735–752. **DOI:** https://doi.org/10.1016/j.neubiorev.2014.07.012, **PMID:** 25155242 25155242 10.1016/j.neubiorev.2014.07.012

[bib71] Moul, C., Cauchi, A., Hawes, D. J., Brennan, J., & Dadds, M. R. (2015). Differentiating autism spectrum disorder and overlapping psychopathology with a brief version of the social responsiveness scale. Child Psychiatry and Human Development, 46(1), 108–117. **DOI:** https://doi.org/10.1007/s10578-014-0456-4, **PMID:** 24604214 24604214 10.1007/s10578-014-0456-4

[bib72] Murphy, C., Jefferies, E., Rueschemeyer, S. A., Sormaz, M., Wang, H. T., Margulies, D. S., & Smallwood, J. (2018). Distant from input: Evidence of regions within the default mode network supporting perceptually-decoupled and conceptually-guided cognition. NeuroImage, 171, 393–401. **DOI:** https://doi.org/10.1016/j.neuroimage.2018.01.017, **PMID:** 29339310, **PMCID:** PMC5883322 29339310 10.1016/j.neuroimage.2018.01.017 PMC5883322

[bib73] Naik, S., Banerjee, A., Bapi, R. S., Deco, G., & Roy, D. (2017). Metastability in senescence. Trends in Cognitive Sciences, 21(7), 509–521. **DOI:** https://doi.org/10.1016/j.tics.2017.04.007, **PMID:** 28499740 28499740 10.1016/j.tics.2017.04.007

[bib74] Naik, S., Subbareddy, O., Banerjee, A., Roy, D., & Bapi, R. S. (2017). Metastability of cortical BOLD signals in maturation and senescence. In 2017 International Joint Conference on Neural Networks (IJCNN) (pp. 4564–4570). IEEE. 10.1109/IJCNN.2017.7966435

[bib75] Nair, A., Treiber, J. M., Shukla, D. K., Shih, P., & Müller, R. A. (2013). Impaired thalamocortical connectivity in autism spectrum disorder: A study of functional and anatomical connectivity. Brain, 136(6), 1942–1955. **DOI:** https://doi.org/10.1093/brain/awt079, **PMID:** 23739917, **PMCID:** PMC3673456 23739917 10.1093/brain/awt079 PMC3673456

[bib76] Noel, J. P., De Niear, M. A., Stevenson, R., Alais, D., & Wallace, M. T. (2017). Atypical rapid audio-visual temporal recalibration in autism spectrum disorders. Autism Research, 10(1), 121–129. **DOI:** https://doi.org/10.1002/aur.1633, **PMID:** 27156926 27156926 10.1002/aur.1633 PMC10791168

[bib77] Nomi, J. S., Bolt, T. S., Ezie, C. C., Uddin, L. Q., & Heller, A. S. (2017). Moment-to-moment BOLD signal variability reflects regional changes in neural flexibility across the lifespan. Journal of Neuroscience, 37(22), 5539–5548. **DOI:** https://doi.org/10.1523/JNEUROSCI.3408-16.2017, **PMID:** 28473644, **PMCID:** PMC5452342 28473644 10.1523/JNEUROSCI.3408-16.2017 PMC5452342

[bib78] Nomi, J. S., & Uddin, L. Q. (2015). Developmental changes in large-scale network connectivity in autism. NeuroImage: Clinical, 7, 732–741. **DOI:** https://doi.org/10.1016/j.nicl.2015.02.024, **PMID:** 25844325, **PMCID:** PMC4375789 25844325 10.1016/j.nicl.2015.02.024 PMC4375789

[bib79] Nomi, J. S., Vij, S. G., Dajani, D. R., Steimke, R., Damaraju, E., Rachakonda, S., … Uddin, L. Q. (2017). Chronnectomic patterns and neural flexibility underlie executive function. NeuroImage, 147, 861–871. **DOI:** https://doi.org/10.1016/j.neuroimage.2016.10.026, **PMID:** 27777174, **PMCID:** PMC5303676 27777174 10.1016/j.neuroimage.2016.10.026 PMC5303676

[bib80] Oldham, S., & Fornito, A. (2019). The development of brain network hubs. Developmental Cognitive Neuroscience, 36, 100607. **DOI:** https://doi.org/10.1016/j.dcn.2018.12.005, **PMID:** 30579789, **PMCID:** PMC6969262 30579789 10.1016/j.dcn.2018.12.005 PMC6969262

[bib81] Olson, L. A., Mash, L. E., Linke, A., Fong, C. H., Müller, R. A., & Fishman, I. (2020). Sex-related patterns of intrinsic functional connectivity in children and adolescents with autism spectrum disorders. Autism, 1362361320938194. **DOI:** https://doi.org/10.1177/1362361320938194, **PMID:** 32689820, **PMCID:** PMC7541740 10.1177/1362361320938194 PMC7541740 32689820

[bib82] Ostrolenk, A., Bao, V. A., Mottron, L., Collignon, O., & Bertone, A. (2019). Reduced multisensory facilitation in adolescents and adults on the autism spectrum. Scientific Reports, 9(1), 1–9. **DOI:** https://doi.org/10.1038/s41598-019-48413-9, **PMID:** 31427634, **PMCID:** PMC6700191 31427634 10.1038/s41598-019-48413-9 PMC6700191

[bib83] Padmanabhan, A., Lynch, C. J., Schaer, M., & Menon, V. (2017). The default mode network in autism. Biological Psychiatry: Cognitive Neuroscience and Neuroimaging, 2(6), 476–486. **DOI:** https://doi.org/10.1016/j.bpsc.2017.04.004, **PMID:** 29034353, **PMCID:** PMC5635856 29034353 10.1016/j.bpsc.2017.04.004 PMC5635856

[bib84] Pelphrey, K. A., Morris, J. P., & McCarthy, G. (2005). Neural basis of eye gaze processing deficits in autism. Brain, 128(5), 1038–1048. **DOI:** https://doi.org/10.1093/brain/awh404, **PMID:** 15758039 15758039 10.1093/brain/awh404

[bib85] Pillai, A. S., & Jirsa, V. K. (2017). Symmetry breaking in space-time hierarchies shapes brain dynamics and behavior. Neuron, 94(5), 1010–1026. **DOI:** https://doi.org/10.1016/j.neuron.2017.05.013, **PMID:** 28595045 28595045 10.1016/j.neuron.2017.05.013

[bib86] Plaisted, K., O’Riordan, M., & Baron-Cohen, S. (1998). Enhanced visual search for a conjunctive target in autism: A research note. Journal of Child Psychology and Psychiatry, 39(5), 777–783. **DOI:** https://doi.org/10.1111/1469-7610.00376, https://doi.org/10.1017/S0021963098002613, **PMID:** 9690940 9690940

[bib87] Preti, M. G., Bolton, T. A., & Van De Ville, D. (2017). The dynamic functional connectome: State-of-the-art and perspectives. NeuroImage, 160, 41–54. **DOI:** https://doi.org/10.1016/j.neuroimage.2016.12.061, **PMID:** 28034766 28034766 10.1016/j.neuroimage.2016.12.061

[bib88] Preti, M. G., & Van De Ville, D. (2019). Decoupling of brain function from structure reveals regional behavioral specialization in humans. Nature Communications, 10(1), 1–7. **DOI:** https://doi.org/10.1038/s41467-019-12765-7, **PMID:** 31628329, **PMCID:** PMC6800438 10.1038/s41467-019-12765-7 PMC6800438 31628329

[bib89] Rashid, B., Blanken, L. M., Muetzel, R. L., Miller, R., Damaraju, E., Arbabshirani, M. R., … Tiemeier, H. (2018). Connectivity dynamics in typical development and its relationship to autistic traits and autism spectrum disorder. Human Brain Mapping, 39(8), 3127–3142. **DOI:** https://doi.org/10.1002/hbm.24064, **PMID:** 29602272, **PMCID:** PMC6045960 29602272 10.1002/hbm.24064 PMC6045960

[bib90] Raut, R. V., Snyder, A. Z., & Raichle, M. E. (2020). Hierarchical dynamics as a macroscopic organizing principle of the human brain. Proceedings of the National Academy of Sciences, 117(34), 20890–20897. **DOI:** https://doi.org/10.1073/pnas.2003383117, **PMID:** 32817467 10.1073/pnas.2003383117 PMC7456098 32817467

[bib91] Ray, D., Hajare, N., Roy, D., & Banerjee, A. (2020). Large-scale functional integration, rather than functional dissociation along dorsal and ventral streams, underlies visual perception and action. Journal of Cognitive Neuroscience, 32(5), 847–861. **DOI:** https://doi.org/10.1162/jocn_a_01527, **PMID:** 31933430 31933430 10.1162/jocn_a_01527

[bib92] Reijmer, Y. D., Schultz, A. P., Leemans, A., O’Sullivan, M. J., Gurol, M. E., Sperling, R., … Hedden, T. (2015). Decoupling of structural and functional brain connectivity in older adults with white matter hyperintensities. NeuroImage, 117, 222–229. **DOI:** https://doi.org/10.1016/j.neuroimage.2015.05.054, **PMID:** 26025290, **PMCID:** PMC4511724 26025290 10.1016/j.neuroimage.2015.05.054 PMC4511724

[bib93] Reiter, M. A., Mash, L. E., Linke, A. C., Fong, C. H., Fishman, I., & Müller, R. A. (2019). Distinct patterns of atypical functional connectivity in lower-functioning autism. Biological Psychiatry: Cognitive Neuroscience and Neuroimaging, 4(3), 251–259. **DOI:** https://doi.org/10.1016/j.bpsc.2018.08.009, **PMID:** 30343132, **PMCID:** PMC7202917 30343132 10.1016/j.bpsc.2018.08.009 PMC7202917

[bib94] Robertson, C. E., & Baron-Cohen, S. (2017). Sensory perception in autism. Nature Reviews Neuroscience, 18(11), 671–684. **DOI:** https://doi.org/10.1038/nrn.2017.112, **PMID:** 28951611 28951611 10.1038/nrn.2017.112

[bib95] Robertson, C. E., Martin, A., Baker, C. I., & Baron-Cohen, S. (2012). Atypical integration of motion signals in autism spectrum conditions. PLoS ONE, 7(11), e48173. **DOI:** https://doi.org/10.1371/journal.pone.0048173, **PMID:** 23185249, **PMCID:** PMC3502435 23185249 10.1371/journal.pone.0048173 PMC3502435

[bib96] Rosenthal, M., Wallace, G. L., Lawson, R., Wills, M. C., Dixon, E., Yerys, B. E., & Kenworthy, L. (2013). Impairments in real-world executive function increase from childhood to adolescence in autism spectrum disorders. Neuropsychology, 27(1), 13–18. **DOI:** https://doi.org/10.1037/a0031299, **PMID:** 23356593, **PMCID:** PMC4747021 23356593 10.1037/a0031299 PMC4747021

[bib97] Saggar, M., Sporns, O., Gonzalez-Castillo, J., Bandettini, P. A., Carlsson, G., Glover, G., & Reiss, A. L. (2018). Towards a new approach to reveal dynamical organization of the brain using topological data analysis. Nature Communications, 9(1), 1–14. **DOI:** https://doi.org/10.1038/s41467-018-03664-4, **PMID:** 29643350, **PMCID:** PMC5895632 10.1038/s41467-018-03664-4 PMC5895632 29643350

[bib98] Sahoo, B., Pathak, A., Deco, G., Banerjee, A., & Roy, D. (2020). Lifespan associated global patterns of coherent neural communication. NeuroImage, 216, 116824. **DOI:** https://doi.org/10.1016/j.neuroimage.2020.116824, **PMID:** 32289459 32289459 10.1016/j.neuroimage.2020.116824

[bib99] Schmitz, N., Rubia, K., Daly, E., Smith, A., Williams, S., & Murphy, D. G. (2006). Neural correlates of executive function in autistic spectrum disorders. Biological Psychiatry, 59(1), 7–16. **DOI:** https://doi.org/10.1016/j.biopsych.2005.06.007, **PMID:** 16140278 16140278 10.1016/j.biopsych.2005.06.007

[bib100] Seghier, M. L., & Price, C. J. (2018). Interpreting and utilising intersubject variability in brain function. Trends in Cognitive Sciences, 22(6), 517–530. **DOI:** https://doi.org/10.1016/j.tics.2018.03.003, **PMID:** 29609894, **PMCID:** PMC5962820 29609894 10.1016/j.tics.2018.03.003 PMC5962820

[bib101] Shafritz, K. M., Dichter, G. S., Baranek, G. T., & Belger, A. (2008). The neural circuitry mediating shifts in behavioral response and cognitive set in autism. Biological Psychiatry, 63(10), 974–980. **DOI:** https://doi.org/10.1016/j.biopsych.2007.06.028, **PMID:** 17916328, **PMCID:** PMC2599927 17916328 10.1016/j.biopsych.2007.06.028 PMC2599927

[bib102] Shine, J. M. (2019). Neuromodulatory influences on integration and segregation in the brain. Trends in Cognitive Sciences, 23(7), 572–583. **DOI:** https://doi.org/10.1016/j.tics.2019.04.002, **PMID:** 31076192 31076192 10.1016/j.tics.2019.04.002

[bib103] Supekar, K., Uddin, L. Q., Khouzam, A., Phillips, J., Gaillard, W. D., Kenworthy, L. E., … Menon, V. (2013). Brain hyperconnectivity in children with autism and its links to social deficits. Cell Reports, 5(3), 738–747. **DOI:** https://doi.org/10.1016/j.celrep.2013.10.001, **PMID:** 24210821, **PMCID:** PMC3894787 24210821 10.1016/j.celrep.2013.10.001 PMC3894787

[bib104] Surampudi, S. G., Misra, J., Deco, G., Bapi, R. S., Sharma, A., & Roy, D. (2019). Resting state dynamics meets anatomical structure: Temporal multiple kernel learning (tMKL) model. NeuroImage, 184, 609–620. **DOI:** https://doi.org/10.1016/j.neuroimage.2018.09.054, **PMID:** 30267857 30267857 10.1016/j.neuroimage.2018.09.054

[bib105] Uddin, L. Q. (2021). Brain mechanisms supporting flexible cognition and behaviour in adolescents with autism spectrum disorder. Biological Psychiatry, 89(2), 172–183. **DOI:** https://doi.org/10.1016/j.biopsych.2020.05.010, **PMID:** 32709415 32709415 10.1016/j.biopsych.2020.05.010 PMC7677208

[bib106] Uddin, L. Q., Supekar, K., Lynch, C. J., Khouzam, A., Phillips, J., Feinstein, C., … Menon, V. (2013). Salience network–based classification and prediction of symptom severity in children with autism. JAMA Psychiatry, 70(8), 869–879. **DOI:** https://doi.org/10.1001/jamapsychiatry.2013.104, **PMID:** 23803651, **PMCID:** PMC3951904 23803651 10.1001/jamapsychiatry.2013.104 PMC3951904

[bib107] Uddin, L. Q., Supekar, K., Lynch, C. J., Cheng, K. M., Odriozola, P., Maria E., Barth, M. E., … Menon, V. (2015). Brain state differentiation and behavioral inflexibility in autism. Cerebral Cortex, 25(12), 4740–4747. **DOI:** https://doi.org/10.1093/cercor/bhu161, **PMID:** 25073720, **PMCID:** PMC4635916 25073720 10.1093/cercor/bhu161 PMC4635916

[bib108] van den Heuvel, M. P., Kahn, R. S., Goñi, J., & Sporns, O. (2012). High-cost, high-capacity backbone for global brain communication. Proceedings of the National Academy of Sciences, 109(28), 11372–11377. **DOI:** https://doi.org/10.1073/pnas.1203593109, **PMID:** 22711833, **PMCID:** PMC3396547 10.1073/pnas.1203593109 PMC3396547 22711833

[bib109] Vázquez-Rodríguez, B., Suárez, L. E., Markello, R. D., Shafiei, G., Paquola, C., Hagmann, P., … Misic, B. (2019). Gradients of structure–function tethering across neocortex. Proceedings of the National Academy of Sciences, 116(42), 21219–21227. **DOI:** https://doi.org/10.1073/pnas.1903403116, **PMID:** 31570622, **PMCID:** PMC6800358 10.1073/pnas.1903403116 PMC6800358 31570622

[bib110] Vidaurre, D., Smith, S. M., & Woolrich, M. W. (2017). Brain network dynamics are hierarchically organized in time. Proceedings of the National Academy of Sciences, 114(48), 12827–12832. **DOI:** https://doi.org/10.1073/pnas.1705120114, **PMID:** 29087305, **PMCID:** PMC5715736 10.1073/pnas.1705120114 PMC5715736 29087305

[bib111] Wang, P., Kong, R., Kong, X., Liégeois, R., Orban, C., Deco, G., … Yeo, B. T. (2019). Inversion of a large-scale circuit model reveals a cortical hierarchy in the dynamic resting human brain. Science Advances, 5(1), eaat7854. **DOI:** https://doi.org/10.1126/sciadv.aat7854, **PMID:** 30662942, **PMCID:** PMC6326747 30662942 10.1126/sciadv.aat7854 PMC6326747

[bib112] Wang, S., Jiang, M., Duchesne, X. M., Laugeson, E. A., Kennedy, D. P., Adolphs, R., & Zhao, Q. (2015). Atypical visual saliency in autism spectrum disorder quantified through model-based eye tracking. Neuron, 88(3), 604–616. **DOI:** https://doi.org/10.1016/j.neuron.2015.09.042, **PMID:** 26593094, **PMCID:** PMC4662072 26593094 10.1016/j.neuron.2015.09.042 PMC4662072

[bib113] Watanabe, T., & Rees, G. (2017). Brain network dynamics in high-functioning individuals with autism. Nature Communications, 8(1), 1–14. **DOI:** https://doi.org/10.1038/ncomms16048, **PMID:** 28677689, **PMCID:** PMC5504272 10.1038/ncomms16048 PMC5504272 28677689

[bib114] Watanabe, T., Rees, G., & Masuda, N. (2019). Atypical intrinsic neural timescale in autism. eLife, 8, e42256. **DOI:** https://doi.org/10.7554/eLife.42256, **PMID:** 30717827, **PMCID:** PMC6363380 30717827 10.7554/eLife.42256 PMC6363380

[bib115] Xia, C. H., Ma, Z., Ciric, R., Gu, S., Betzel, R. F., Kaczkurkin, A. N., … Cui, Z. (2018). Linked dimensions of psychopathology and connectivity in functional brain networks. Nature Communications, 9(1), 1–14. **DOI:** https://doi.org/10.1038/s41467-018-05317-y, **PMID:** 30068943, **PMCID:** PMC6070480 10.1038/s41467-018-05317-y PMC6070480 30068943

[bib116] Yao, Z., Hu, B., Xie, Y., Zheng, F., Liu, G., Chen, X., … Zheng, W. (2016). Resting-state time-varying analysis reveals aberrant variations of functional connectivity in autism. Frontiers in Human Neuroscience, 10, 463. **DOI:** https://doi.org/10.3389/fnhum.2016.00463, **PMID:** 27695408, **PMCID:** PMC5025431 27695408 10.3389/fnhum.2016.00463 PMC5025431

